# SARS‐CoV‐2 Evolution: Immune Dynamics, Omicron Specificity, and Predictive Modeling in Vaccinated Populations

**DOI:** 10.1002/advs.202402639

**Published:** 2024-08-29

**Authors:** Xiaohan Zhang, Mansheng Li, Nana Zhang, Yunhui Li, Fei Teng, Yongzhe Li, Xiaomei Zhang, Xingming Xu, Haolong Li, Yunping Zhu, Yumin Wang, Yan Jia, Chengfeng Qin, Bingwei Wang, Shubin Guo, Yajie Wang, Xiaobo Yu

**Affiliations:** ^1^ State Key Laboratory of Medical Proteomics Beijing Proteome Research Center National Center for Protein Sciences‐Beijing (PHOENIX Center) Beijing Institute of Lifeomics Beijing 102206 China; ^2^ School of Medicine Nanjing University of Chinese Medicine Nanjing 210023 China; ^3^ Department of Virology State Key Laboratory of Pathogen and Biosecurity Beijing Institute of Microbiology and Epidemiology Academy of Military Medical Sciences Beijing 100071 China; ^4^ Department of Clinical Laboratory Beijing Ditan Hospital Capital Medical University Beijing 100015 China; ^5^ Emergency Medicine Clinical Research Center Beijing Chao‐Yang Hospital Capital Medical University Beijing Key Laboratory of Cardiopulmonary Cerebral Resuscitation Beijing 100020 China; ^6^ Department of Clinical Laboratory Peking Union Medical College Hospital Chinese Academy of Medical Science & Peking Union Medical College Beijing 100730 China; ^7^ The First Affiliated Hospital of Wenzhou Medical University Wenzhou 325000 China; ^8^ ProteomicsEra Medical Co. Ltd. Beijing 102206 China

**Keywords:** evolution, neutralizing antibody, prevalence, proteomics, SARS‐CoV‐2

## Abstract

Host immunity is central to the virus's spread dynamics, which is significantly influenced by vaccination and prior infection experiences. In this work, we analyzed the co‐evolution of SARS‐CoV‐2 mutation, angiotensin‐converting enzyme 2 (ACE2) receptor binding, and neutralizing antibody (NAb) responses across various variants in 822 human and mice vaccinated with different non‐Omicron and Omicron vaccines is analyzed. The link between vaccine efficacy and vaccine type, dosing, and post‐vaccination duration is revealed. The classification of immune protection against non‐Omicron and Omicron variants is co‐evolved with genetic mutations and vaccination. Additionally, a model, the Prevalence Score (P‐Score) is introduced, which surpasses previous algorithm‐based models in predicting the potential prevalence of new variants in vaccinated populations. The hybrid vaccination combining the wild‐type (WT) inactivated vaccine with the Omicron BA.4/5 mRNA vaccine may provide broad protection against both non‐Omicron variants and Omicron variants, albeit with EG.5.1 still posing a risk. In conclusion, these findings enhance understanding of population immunity variations and provide valuable insights for future vaccine development and public health strategies.

## Graphical abstract

This study analyzes the co‐evolution of SARS‐CoV‐2 mutation, ACE2 receptor binding, and NAb responses across various variants using SARS‐CoV‐2 broad neutralizing antibody (bNAb) assay. The results reveal the link between vaccine efficacy and vaccine type, dosing, and the hybrid vaccination combining the WT inactivated vaccine with the Omicron BA.4/5 mRNA vaccine may provide broad protection against both non‐Omicron variants and Omicron variants.

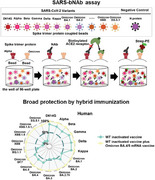

## 1 Introduction

As of January 2024, the Global Initiative on Sharing Avian Influenza Database (GISAID) has documented over sixteen million variants of the SARS‐CoV‐2 virus genome.^[^ ^1^ ^]^ This virus has undergone significant adaptation through mutation and evolution, enhancing its transmission and immune evasion capabilities as it coexists with the human population.^[^ ^2^, ^3^, ^4^ ^]^

The spike protein of SARS‐CoV‐2 is a primary target for most COVID‐19 vaccines due to its crucial role in enabling the virus to enter host cells. Within this protein, the receptor‐binding domain (RBD) specifically binds to the angiotensin‐converting enzyme 2 (ACE2) receptor in human cells, facilitating viral entry and infection.^[^ ^5^, ^6^ ^]^ Mutations within the RBD can significantly impact the virus's ability to infect and evade the immune system. mRNA vaccines, such as those developed by Pfizer‐BioNTech and Moderna,^[^ ^7^, ^8^ ^]^ encode the spike protein, while a thermostable vaccine from China encodes the RBD protein.^[^ ^9^ ^]^

The nucleocapsid (N) protein is a key structural component of SARS‐CoV‐2, essential for packaging and protecting the viral RNA genome. It consists of two main domains: the N‐terminal RNA‐binding domain (NTD) and the C‐terminal dimerization domain (CTD), both of which are vital for RNA binding and oligomerization.^[^ ^10^, ^11^ ^]^ This protein is highly conserved across coronaviruses, making it an attractive target for broad‐spectrum coronavirus vaccines.^[^ ^12^ ^]^ Various research groups and companies are exploring the N protein as a potential vaccine target. For instance, Gao et al. demonstrated in a study that an N protein‐based vaccine elicited robust T‐cell responses and provided protection in a mouse model.^[^ ^13^ ^]^ Another study by Zeng et al. indicated that an N protein vaccine could trigger both humoral and cellular immune responses in non‐human primates.^[^ ^14^ ^]^ However, unlike the S protein, the N protein does not elicit strong neutralizing antibody (NAb) responses, which are crucial for preventing viral entry into host cells. This necessitates the use of adjuvants or combination with other antigens to enhance efficacy. Furthermore, studies have suggested that incorporating NTDs into the immunogen of COVID‐19 vaccines can broaden the neutralizing epitope and decrease the likelihood of immune escape. Additionally, RBD NAb and NTD NAb exhibit significant synergistic effects in neutralizing the live novel coronavirus.^[^ ^15^ ^]^

In the realm of COVID‐19 vaccine development, inactivated vaccines are produced by cultivating the virus in a controlled environment and then deactivating it using substances like formaldehyde or beta‐propiolactone, rendering it unable to replicate. This method preserves the virus's structure, enabling the immune system to recognize and combat it without causing illness. Several inactivated COVID‐19 vaccines, such as CoronaVac (from Sinovac), BBIBP‐CorV (from Sinopharm), and Covaxin (from Bharat Biotech), have been developed and authorized for emergency use.

The Omicron variant, characterized by multiple mutations in the spike protein, particularly in the RBD, has the potential to reduce the effectiveness of vaccines designed for earlier strains of the virus.^[^ ^15^ ^]^ Among the strategies developed to combat the virus, mRNA vaccines have proven to be highly effective in expressing RBD protein, especially in addressing the mutations present in Omicron variants.^[^ ^9^, ^16^, ^17^ ^]^ This is crucial to produce specific NAbs to Omicron variants with high homology.^[^ ^17^ ^]^ A vaccine candidate targeting the Omicron variant was successfully created and demonstrated to stimulate an immune response in mice within a short period of 32 days, showcasing the rapid nature of the mRNA‐LNP technology platform.^[^ ^18^ ^]^ Furthermore, research on the mRNA vaccine against BA.4/5 has shown that a single booster dose of the BA.4/5 monovalent mRNA vaccine can generate protective antibodies against various circulating strains, including the XBB.1 lineage, across different populations.^[^ ^16^ ^]^

Regarding efficacy and adverse effects, a meta‐analysis by Lv et al. analyzed 22 vaccines using phase III randomized controlled trial data up to September 30, 2023. The study found no significant differences in efficacy among vaccine types in preventing symptomatic SARS‐CoV‐2 infection, though mRNA vaccines showed a trend toward higher efficacy with a surface under the cumulative ranking curve (SUCRA) value of 0.09. BNT162b2 exhibited the highest efficacy (SUCRA value: 0.02), followed by mRNA‐1273, with BNT162b2 also demonstrating the highest efficacy in the elderly population (SUCRA value: 0.08). In terms of safety, inactivated vaccines may be considered the safest, with a SUCRA value of 0.16, and BIV1‐CovIran displaying the highest safety index (SUCRA value: 0.13).^[^ ^19^ ^]^

Despite the global vaccination efforts covering 13.34 billion people, breakthrough infections of the SARS‐CoV‐2 virus persist, often leading to long‐term complications,^[^ ^20^, ^21^ ^]^ especially in individuals with underlying health conditions.^[^ ^22^, ^23^ ^]^ Efforts to understand the genetic and antigenic evolution of SARS‐CoV‐2 variants and predict immune evasion have been extensive, employing various algorithms such as PyR0, MLAEP, and EVEscape.^[^ ^24^, ^25^, ^26^, ^27^, ^28^, ^29^ ^]^ Nevertheless, the transmission potential of new variants predicted by these algorithms may be hindered due to the continuous reinforcement of population immunity through vaccination and/or natural infection.^[^ ^24^ ^]^ Furthermore, variations in population immunity among distinct regions may be attributed to differences in vaccine types and vaccination dosages. Nevertheless, there is a dearth of information concerning the adaptive changes in vaccination‐induced immunity in the context of SARS‐CoV‐2 genetic evolution, and the classification of serological responses to different SARS‐CoV‐2 variants induced by various vaccines remains ambiguous.^[^ ^30^, ^31^, ^32^ ^]^

For example, in 2022, Simon‐Loriere et al. proposed a classification of SARS‐CoV‐2 variants into non‐Omicron and Omicron serotypes through phylogenetic analysis of genetic sequences related to SARS‐CoV‐2.^[^ ^30^ ^]^ Tan et al. investigated the antigenic relationships among various SARS‐CoV‐2 variants by analyzing serum samples from individuals who had recovered from primary infections with various SARS‐related viruses. The study's findings categorized these variants into three serotypes: SARS‐CoV‐1, SARS‐CoV‐2 non‐Omicron, and Omicron.^[^ ^33^ ^]^ Hu et al. employed a mouse model to systematically assess the antigenic properties of RBD using mRNA vaccines based on the RBDs of 23 distinct SARS‐CoV‐2 variants. This evaluation resulted in the classification of these variants into five serotypes, comprising two subtypes of non‐Omicron variants and four serotypes associated with diverse Omicron variants.^[^ ^31^ ^]^

To address these challenges, we leveraged a previously established SARS‐CoV‐2 broad neutralizing antibody (bNAb) assay, enabling high‐throughput quantification of serum NAbs against diverse SARS‐CoV‐2 variants. This assay exhibited strong correlations with commercial IgG serological assay (*R* = 0.89), the FDA‐approved cPass sVNT assay (*R* = 0.93), pseudovirus‐based neutralization assay (*R* = 0.96 for D614G variant, *R* = 0.66 for Omicron BA.1 variant) and live virus‐based neutralization assay (*R* = 0.79 for D614G variant, *R* = 0.64 for Omicron BA.1 variant).^[^ ^34^ ^]^ In this retrospective analysis, we examined the immunological responses of 822 sera to diverse SARS‐CoV‐2 variants (D614G, Alpha, Beta, Gamma, Delta, Kappa, BA.1, BA.2, BA.3, BA.4, BA.5, BF.7, BA.2.75, BQ.1.1, XBB, XBB.1.5, XBB.1.9.1, and EG.5.1), considering the vaccination history (inactivated, recombinant or mRNA vaccines) and different doses received by human and mouse model. The study utilized the SARS‐CoV‐2 bNAb assay, pseudovirus and live virus‐based neutralization assays, and integrated data to comprehensively profile vaccination immunity and understand the co‐evolution of SARS‐CoV‐2 genetic variation, ACE2 receptor binding, and vaccination immunity.

Our findings elucidate distinct immunological classifications regulated by vaccination and provide valuable insights into the adaptive changes in vaccination immunity concerning SARS‐CoV‐2 genetic evolution. We also introduce a predictive model, the Prevalence Score (*P*‐Score), incorporating RBD mutation rates, ACE2 receptor binding, and proteomic data related to immune evasion. This model exhibits the potential to evaluate the prevalence risk of emerging SARS‐CoV‐2 variants in real‐world scenarios.

## 2 Results

### 2.1 Profiling of SARS‐CoV‐2 Serum NAbs in an Inactivated or Recombinant Vaccine Administration Cohort

**Figure 1:**
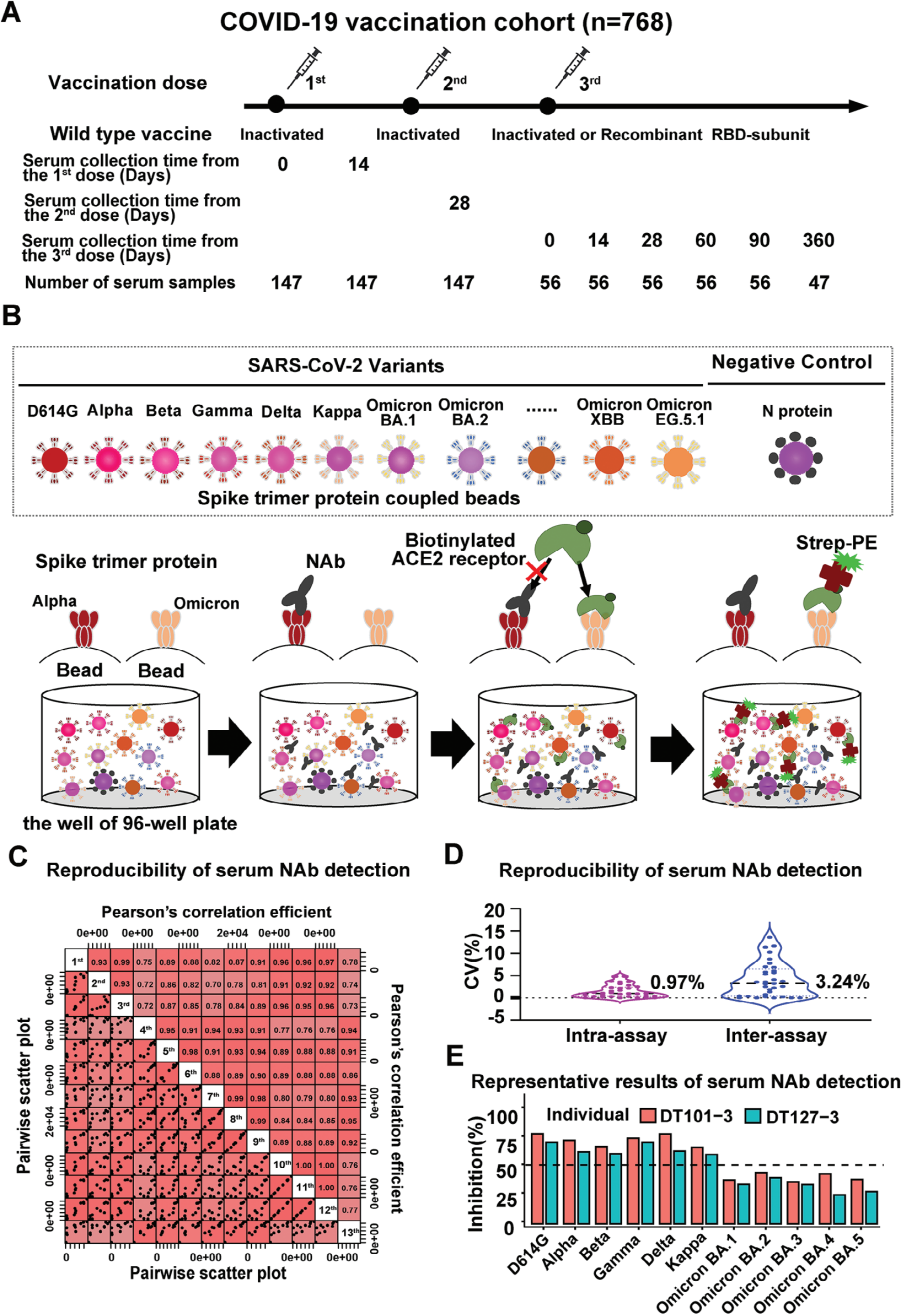
Determination of serum NAbs in an inactivated or recombinant vaccine administration cohort using SARS‐CoV‐2 bNAb Assay. A) Depicts the timeline of serum sample collection from vaccinated participants. B) Provides a schematic representation of the NAb detection process using the SARS‐CoV‐2 bNAb assay. C) Scatter plots and Pearson's correlation coefficients for depicting the reproducibility of serum NAb detection between replicates. The bottom‐left half of the panel represents the pairwise scatter plots of the replicates and the top‐right half of the panel depicts the pairwise Pearson's correlation coefficients from the same comparison. D) Shows the assay variation within an experiment and across different experiments. E) Displays the representative outcomes of NAb cross‐reaction to diverse SARS‐CoV‐2 variants in individuals 28 days after receiving the third dose of inactivated vaccination. The dashed line of the 50% inhibition rate is provided as a reference.

A total of 768 sera from 203 individuals, who received the first, second, and third doses of inactivated whole‐virion SARS‐CoV‐2 vaccine or recombinant RBD vaccine for the Wuhan strain were collected in accordance with the Declaration of Helsinki (**Figure** **1**A; Tables S1, S2, Supporting Information). Serum NAbs against eleven SARS‐CoV‐2 variants (D614G, Alpha, Beta, Gamma, Delta, Kappa, Omicron BA.1, BA.2, BA.3, BA.4, and BA.5) were assessed using SARS‐CoV‐2 bNAb assay.^[^ ^34^ ^]^ Briefly, trimeric spike proteins from various SARS‐CoV‐2 variants were coupled to fluorescence‐encoded magnetic beads, with N protein serving as the negative control. Preceding the assay, these beads were incubated with clinical serum samples, wherein the binding of serum NAbs to the spike protein hindered its interaction with the biotinylated ACE2 receptor and streptavidin‐phycoerythrin (SA‐PE).^[^ ^34^ ^]^ The inhibition rate (%) was calculated by subtracting the serum data from vaccinated individuals from that of unvaccinated and uninfected individuals (Figure 1B).

The assessment of assay reproducibility revealed r correlations of Spike‐ACE2 binding signals ranging from 0.77 to 0.97 (Figure 1C). The average variation in NAb detection within a single experiment and between different experiments was 0.97% and 3.24%, respectively (Figure 1D). A representative detection result is depicted in Figure 1E, showcasing that the third dose of inactivated or recombinant RBD vaccine administration in two individuals induced cross‐neutralization against various SARS‐CoV‐2 variants, spanning from D614G to Delta, Gamma, Alpha, Beta, Kappa, Omicron BA.2, BA.4, BA, 5, BA.1, and BA.3. These findings align with previous studies,^[^ ^35^, ^36^ ^]^ affirming the reliability and reproducibility of serum NAb detection using our proteomics technology.

Furthermore, we conducted a comparison of serum NAbs between individuals in the COVID‐19 vaccinated group (*n* = 54) and those who experienced a breakthrough infection with the delta variant (*n* = 9). The results revealed that the NAb levels in the breakthrough group were significantly higher than those in the vaccinated group (*p* < 0.001) (Figure S1, Supporting Information), highlighting the effectiveness of our assay in distinguishing between infection and vaccination.

At last, we compared the high‐throughput methods that determine the NAbs in previous studies, including Bead‐based multiplex ACE2‐RBD inhibition assay (RBDCoV‐ACE2), Bio‐Plex Pro Human SARS‐CoV‐2 Neutralization Antibody assay and FDA‐approved cPass sVNT assay that was developed based on the Luminex, Bio‐rad bead‐based array and ELISA platform (Table S3, Supporting Information). The results show that our SARS‐CoV‐2 bNAb assay has the largest panel of SARS‐CoV‐2 variants that can be determined to date.

### 2.2 Landscape Mapping of Vaccination Immunity to SARS‐CoV‐2 Variants by Inactivated or Recombinant Vaccine

**Figure 2:**
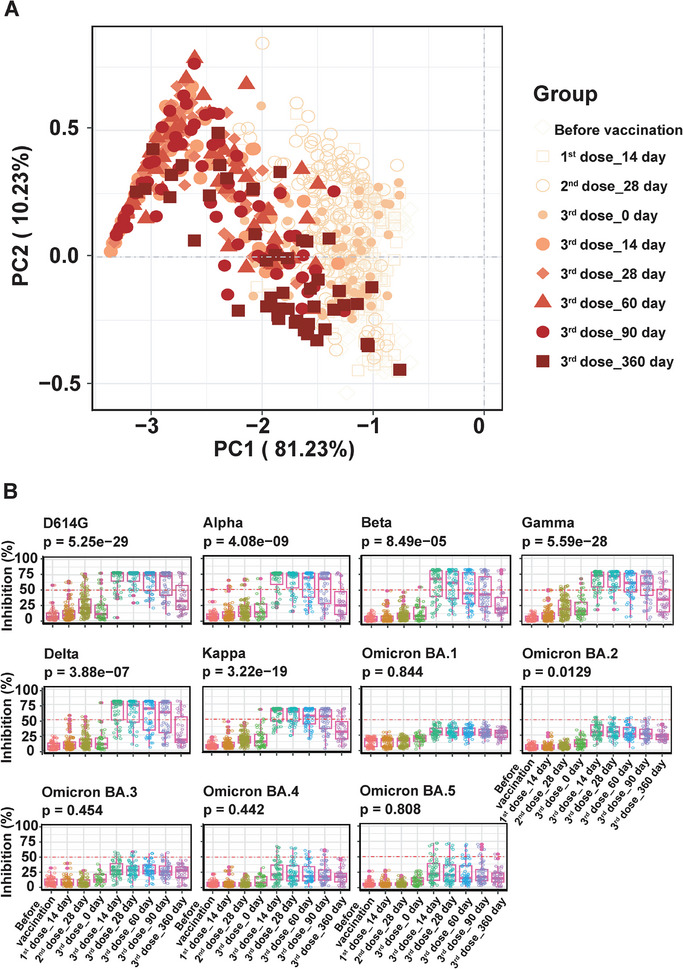
Longitudinal changes of serum NAbs before and after vaccination. A) Presents the principal component analysis (PCA) of vaccination individuals based on their serum NAbs inhibition rate (%) against SARS‐CoV‐2 variants. B) Shows the longitudinal alterations in the inhibition rate (%) of NAbs against various SARS‐CoV‐2 variants before and after different doses of vaccination. The x‐axis indicates the days before and after vaccination, while the *y*‐axis represents the inhibition rate (%) of serum antibodies after subtracting the background obtained from pre‐pandemic serum. The comparison of the mean of the inhibition rate (%) of NAbs against different SARS‐CoV‐2 variants was conducted using ANOVA test. The dashed line of the 50% inhibition rate is provided as a reference.

To comprehend the adaptive changes in humoral immunity driven by vaccination, we employed principal component analysis to visualize individuals without and with the first, second, and third doses (**Figure** **2**A). The results depicted distinct clusters based on vaccination status and doses. Moving from right to left, clusters corresponded to individuals without (prismatic) and with the first (square), second (circle, small solid dot), and third dose (large solid dot, solid prismatic, triangle, solid brown circle, and square). Notably, cluster formation was also influenced by the time (days) post‐vaccination. Specifically, individuals with the third dose were clustered according to the time intervals (0, 14, 28, 60, 90, and 360 days) post‐vaccination, illustrating a leftward movement corresponding to the waxing and waning of immunity.

The longitudinal alterations in cross‐neutralization of serum NAbs to SARS‐CoV‐2 variants are presented in Figure 2B. The results revealed a slight increase in NAb inhibition rate (%) for all SARS‐CoV‐2 variants with the first and second dose vaccinations, notably enhanced by the third dose vaccination. The NAb inhibition rate (%) peaked at 14–28 days and then declined up to day 360 of testing. Among SARS‐CoV‐2 variants, serum NAbs exhibited the highest inhibition against D614G, followed by the Alpha, Delta, Gamma, and Kappa variants, with inhibition rates (%) exceeding 50% until day 90. In contrast, NAbs against Omicron variants (BA.1, BA.2, BA.3, BA.4, and BA.5) remained significantly lower throughout the study period, with an average inhibition rate of ≈30%.

In addition, we analyzed the relationship between the NAb levels and adverse effects (Injection site pain, redness, and swelling) induced by inactivated vaccination with the information that was available in the first‐dose and second‐dose vaccination cohort (Table S2, Supporting Information). The results showed that there has no difference between the two groups with (*n* = 14) and without adverse effects (injection site pain, redness, and swelling) (*n* = 133) (Figure S1, Supporting Information), which is agrees with previous study on BNT162b2 or mRNA‐1273 vaccine.^[^ ^37^ ^]^

### 2.3 Co‐Evolution of SARS‐CoV‐2 Variants and Vaccination Immunity

**Figure 3:**
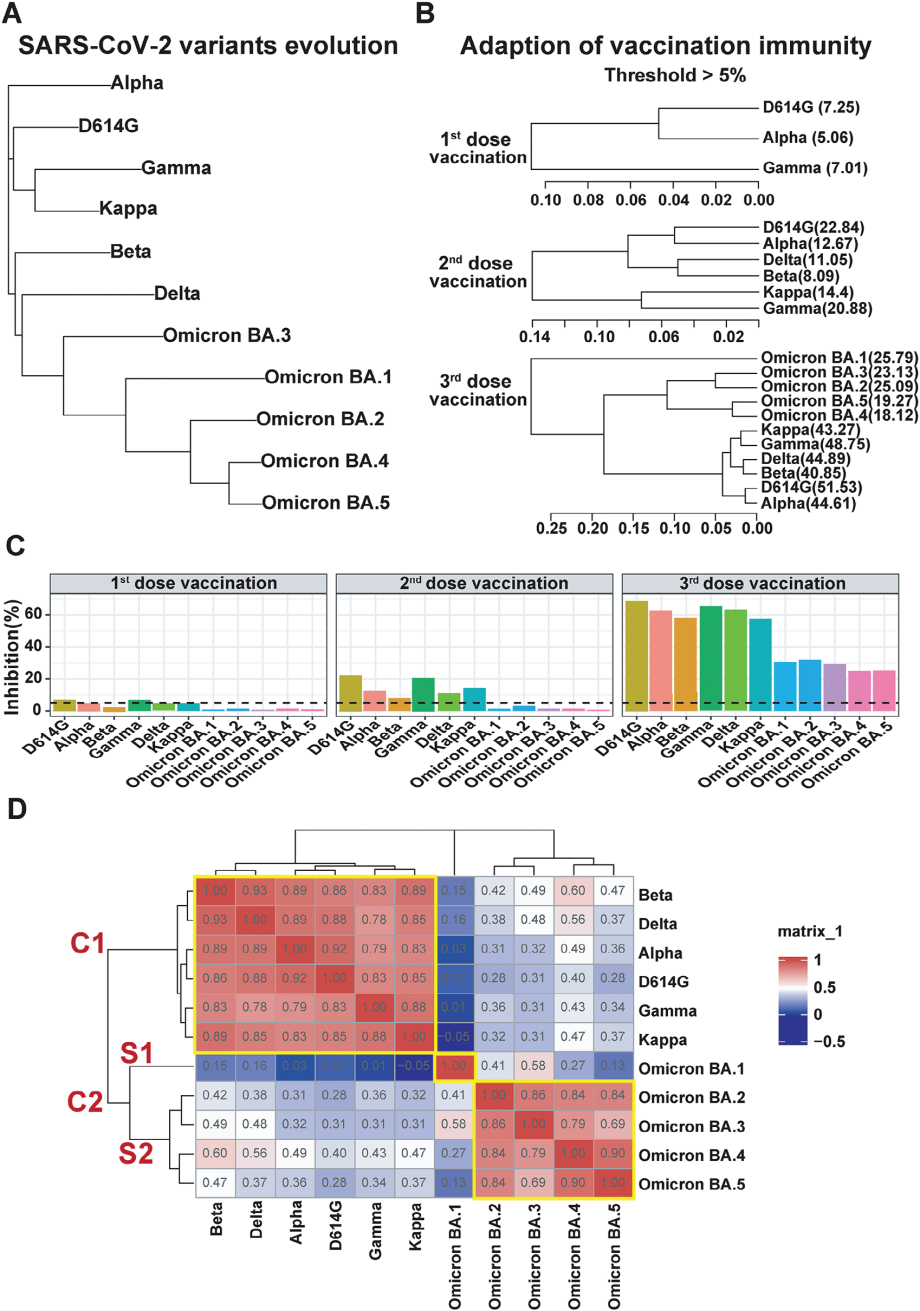
Adaptive changes in vaccination immunity in response to the evolution of SARS‐CoV‐2 variants. A) Depicts the evolutionary trajectory of SARS‐CoV‐2 variants. B) Demonstrates the adaptive evolution of vaccination immunity in the context of SARS‐CoV‐2 variant evolution under the inactivated or recombinant RBD vaccine administration. C) Presents the inhibition rate (%) of vaccination immunity against various SARS‐CoV‐2 non‐Omicron and Omicron variants in individuals who received the first, second, and third vaccine doses. D) Displays the non‐biased hierarchical clustering analysis of vaccination immunity and SARS‐CoV‐2 non‐Omicron and Omicron variants. The non‐biased hierarchical clustering analysis was conducted using Pearson's correlation distance and complete linkage method implemented in the R package Complexheatmap (version 2.16.0).

To investigate the co‐evolution of SARS‐CoV‐2 and vaccination immunity, we performed a homological analysis of SARS‐CoV‐2 variants by conducting multi‐sequence alignments among their spike protein sequences and constructing their phylogenetic tree (**Figure** **3**A). The results reveal a continuous evolution of SARS‐CoV‐2 variants from Alpha, D614G, Gamma, Kappa, Beta, Delta to Omicron BA.3, BA.1, BA.2, BA.4, and BA.5.

Simultaneously, vaccination immunity demonstrated adaptability to changes in vaccination doses. Using a 5% inhibition rate as a threshold, our findings indicate that the first vaccination dose induces serum NAbs against Alpha, D614G, and Gamma. Immunity is further fortified with the booster vaccination, where the second dose generates NAbs against all non‐Omicron variants (Alpha, D614G, Gamma, Kappa, Beta, and Delta). Notably, the third vaccination significantly elevates NAb production, targeting not only non‐Omicron variants but also Omicron variants (BA.1, BA.2, BA.3, BA.4, and BA.5) (Figure 3B,C). Unfortunately, the inhibition rate (%) of NAbs is halved in Omicron variants compared to non‐Omicron variants, highlighting the potential risk of breakthrough infection for vaccinated individuals facing Omicron variants.

### 2.4 Classification of Vaccination Immunity to Non‐Omicron and Omicron Variants

The categorization of SARS‐CoV‐2 serotypes is pivotal for understanding antigenic differences among various variants and developing more targeted diagnostics and effective vaccines for specific populations.^[^ ^30^ ^,^ ^33^ ^,^ ^38^ ^]^ However, the interrelationship among different SARS‐CoV‐2 variants for their cross‐neutralization reactivity in humans remains unclear. To address this gap, we conducted an unbiased hierarchical clustering analysis of NAb responses to diverse SARS‐CoV‐2 variants within the vaccination cohort.^[^ ^39^ ^]^ The results demonstrated that NAb responses to different SARS‐CoV‐2 variants could be clustered into two distinct groups through unbiased hierarchical clustering analysis using Pearson's correlation distance and the complete linkage method (Figure 3D). Notably, the SARS‐CoV‐2 variants were classified into two clusters: the non‐Omicron variants group (D614G, Alpha, Beta, Gamma, Delta, and Kappa) and the Omicron variants group (BA.1‐BA.5).

**Figure 4:**
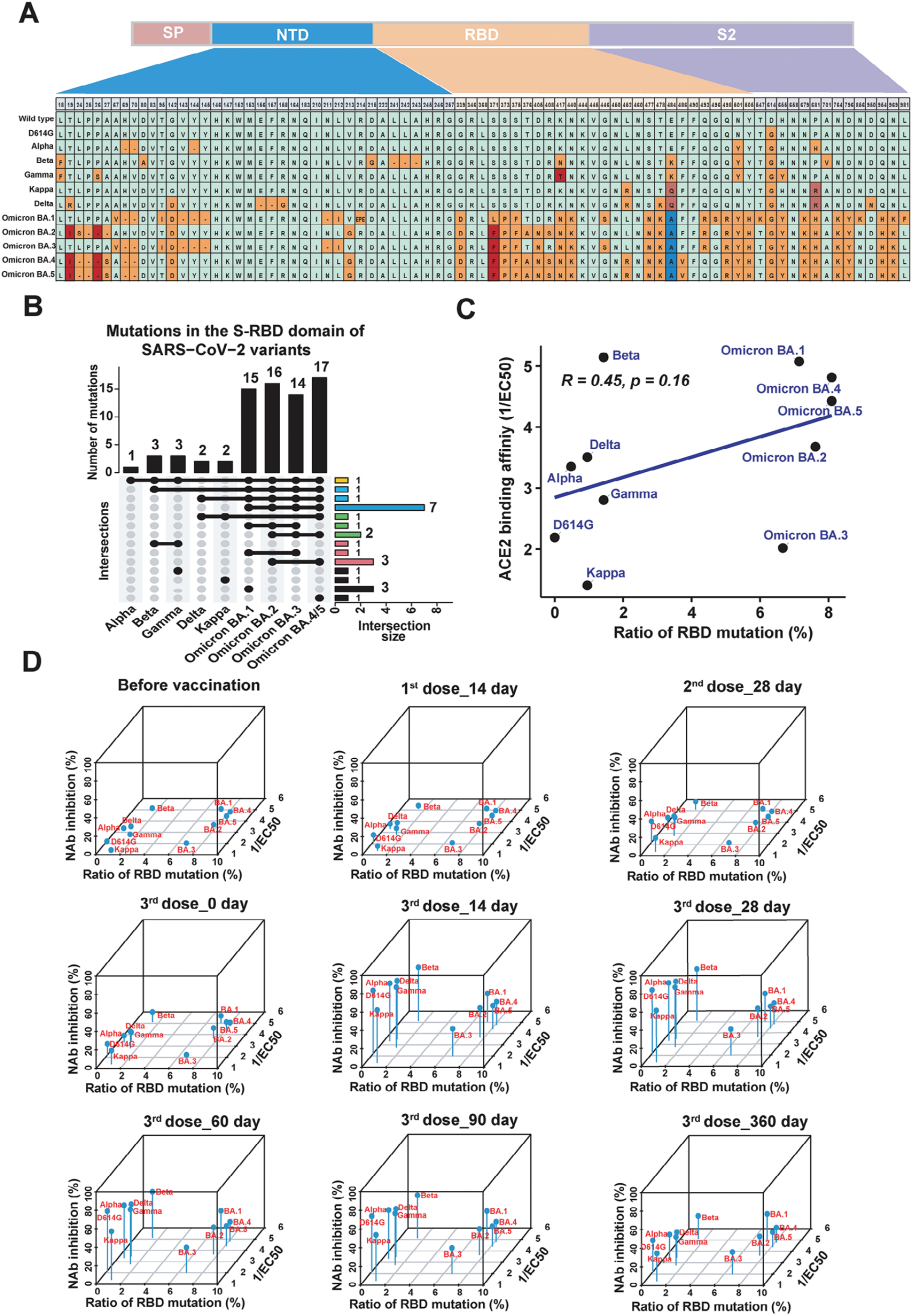
Systematic analysis of interactions among SARS‐CoV‐2 RBD mutation rate, ACE2 receptor binding, and immune evasion in vaccinated cohort. A) Illustrates the mutated amino acids within the RBD of the spike protein in various SARS‐CoV‐2 variants. B) Presents an UpSet plot revealing the number and intersections of mutations located on the S protein RBD of Alpha, Beta, Gamma, Delta, Kappa, BA.1, BA.2, BA.3, BA.4, and BA.5 variants. C) Shows the relationship between Spike‐ACE2 binding affinity and the percentage of mutated amino acids in the RBD domain. D) Illustrates the dynamic profiling of interactions among SARS‐CoV‐2 genetic mutation, ACE2 binding, and immune evasion in a vaccinated cohort.

In Cluster 1, D614G exhibited a robust correlation with other variants, ranging from 0.83 to 0.92. Cluster 2 is further divided into two sub‐clusters, Sub‐cluster 1 (BA.1) and Sub‐cluster 2 (BA.2‐5). Remarkably, BA.1 showed no correlation with any non‐Omicron variants (−0.05–0.16) and a moderate correlation with BA.2‐5 variants (0.13–0.58). This observation aligns with the genetic evolution of SARS‐CoV‐2, where BA.1 serves as the ancestor of Omicron variants (Figures 3A and 4A), corroborating the serotypes identified genetic evolution analysis^[^ ^40^ ^]^ and cross‐neutralization analysis in the convalescent sera from unvaccinated individuals previously infected with SARS‐CoV‐1 and SARS‐CoV‐2 variants.^[^ ^33^ ^]^

Employing D614G and Omicron BA.1 as illustrative examples, we delved deeper into the differential binding of the NAb to non‐Omicron and Omicron variants through molecular dynamics simulations, utilizing the previously identified REGN10933 (Figures S3–S5, Supporting Information).^[^ ^41^, ^42^, ^43^ ^]^ Our simulation outcomes unveiled marked disparities in hydrogen bond formation between the D614G and Omicron BA.1 systems, particularly evident in Figure S2B (Supporting Information). Additionally, notable differences emerged in binding free energies, as detailed in Table S4 and Figure S3 (Supporting Information). Notably, the electrostatic force (Δ*G*_ele_) emerged as the primary determinant in the binding process, with the D614G system exhibiting significantly stronger Δ*G*_ele_ compared to the Omicron BA.1 system. The van der Waals force (Δ*G*_vdw_) ranked second in driving binding, maintaining a similar order of strength. These two forces collectively underpin the superior binding affinity of the D614G system. Despite the D614G system encountering greater hindrance in terms of solvation energy, including polar (Δ*G*_pola_r) and non‐polar (Δ*G*_nonpolar_) components, this did not compromise its overall binding prowess. In terms of total binding energy, the D614G system (Δ*G*_total_ = −73.6229 kcal mol^−1^) demonstrated notably better binding affinity than the Omicron BA.1 system (Δ*G*_total_ = −47.6264 kcal mol^−1^), signifying a tighter binding of REGN10933 to the D614G variant, which rationalizes its higher neutralization potency against this variant compared to Omicron BA.1 (Table S4, Supporting Information).

Furthermore, we conducted energy decomposition calculations for both systems. The energy contributions of individual residues in the D614G system (<−1 kcal mol^−1^) and their corresponding counterparts in the Omicron BA.1 system are presented in Figure S4 (Supporting Information). Residues in the D614G system generally contributed more energy, with Ser477, Tyr53, Tyr453, Leu455, Lys417 (Asn417 in Omicron BA.1), and Gly476 exhibiting the most pronounced differences, each exceeding 2 kcal mol^−1^ in energy contribution. These residues play a pivotal role in maintaining system stability, and their energy disparities can be attributed to residue mutations in the Omicron BA.1 variant, leading to reduced antibody binding and consequently, compromised neutralization efficacy against this variant.

To gain insights into the underlying binding mechanisms, we performed molecular dynamics simulation trajectory cluster analysis, yielding representative conformations for both D614G and Omicron BA.1 systems (Figure S5, Supporting Information). While the binding modes share similarities, notable differences were observed. In the D614G system, specific residues engage in hydrophobic interactions with REGN10933's light and heavy chain residues, alongside hydrogen bonds. Conversely, the Omicron BA.1 system exhibits significantly reduced hydrophobic interactions and hydrogen bonds, primarily due to residue mutations. This reduction in interaction forces ultimately translates to weakened neutralizing efficacy of REGN10933 against the Omicron BA.1 variant.

### 2.5 Interactions among SARS‐CoV‐2 Mutation Rate, ACE2 Receptor Binding, and Serum NAbs in the Context of Vaccination

The significance of genetic evolution, ACE2 receptor binding, and immune evasion in SARS‐CoV‐2 transmission and prevalence has been well‐established.^[^ ^2^, ^44^ ^]^ An examination of genetic evolution reveals a notable increase in mutations within the BA.1 sequence compared to non‐Omicron variants (**Figure** **4**A). As mutations within the RBD are pivotal for immune evasion,^[^ ^2^ ^]^ we conducted an upset plot to visually represent the number and intersections of mutations situated on the S protein RBD. This analysis was performed for both the non‐Omicron variants group and the Omicron variants group (Figure 4B; Figure S5, Supporting Information). The results depict the ranked common and unique mutations observed in the variants. The number of mutations located on the S protein RBD of Omicron variants is substantially higher than that of non‐Omicron variants. The Omicron variants group (BA.1‐BA.5) shares 11 mutations in RBD, including G339D, S373P, S375F, K417N, N440K, S477N, T478K, E484A, Q498R, Y505H, and N501Y. Numerous studies have indicated that Omicron variants can infect individuals recovering from infections by previously prevalent variants.^[^ ^45^ ^]^ These mutations may form the basis for Omicron variants to evade immunity induced by previous infections.

Next, we analyzed the relationship between RBD mutation rate and ACE2 receptor binding obtained by coating the trimeric spike protein from different SARS‐CoV‐2 variants using a bead‐based assay as previously described.^[^ ^34^ ^]^ The results showed that the non‐Omicron variants and Omicron variants clustered together into two distinct groups, and the RBD mutation rate is not correlated with ACE2 binding affinity (Figure 4C). The integrative analysis of RBD mutation rate, ACE2 binding, and vaccination immunity revealed distinct clustering of non‐Omicron and Omicron variants according to the RBD mutation rate. Variants with high RBD mutation rates, high ACE2 binding affinity, and strong immune evasion tended to be prevalent previously (Figure 4D). For example, D614G has a low binding affinity and can be well inhibited by inactivated or recombinant RBD vaccine administration, along with other non‐Omicron variants. In comparison, BA.1, BA.2, BA.4, and BA.5 variants, with high RBD mutation rates, high ACE2 binding affinity, and immune evasion but low NAb levels, showed prevalence in the last two years, except BA.3 with low ACE2 binding affinity. All these results provide direct evidence for the co‐regulation of three critical factors (genetic mutation, ACE2 receptor binding, and immune evasion) in SARS‐CoV‐2 transmission and prevalence, indicating that population immunity induced by inactivated or recombinant RBD vaccine administration may not be sufficient to protect the host against Omicron infection.

### 2.6 Predictive Modeling of SARS‐CoV‐2 Variant Prevalence: Integrating RBD Mutation Rate, ACE2 Receptor Binding, and Immune Evasion

**Figure 5:**
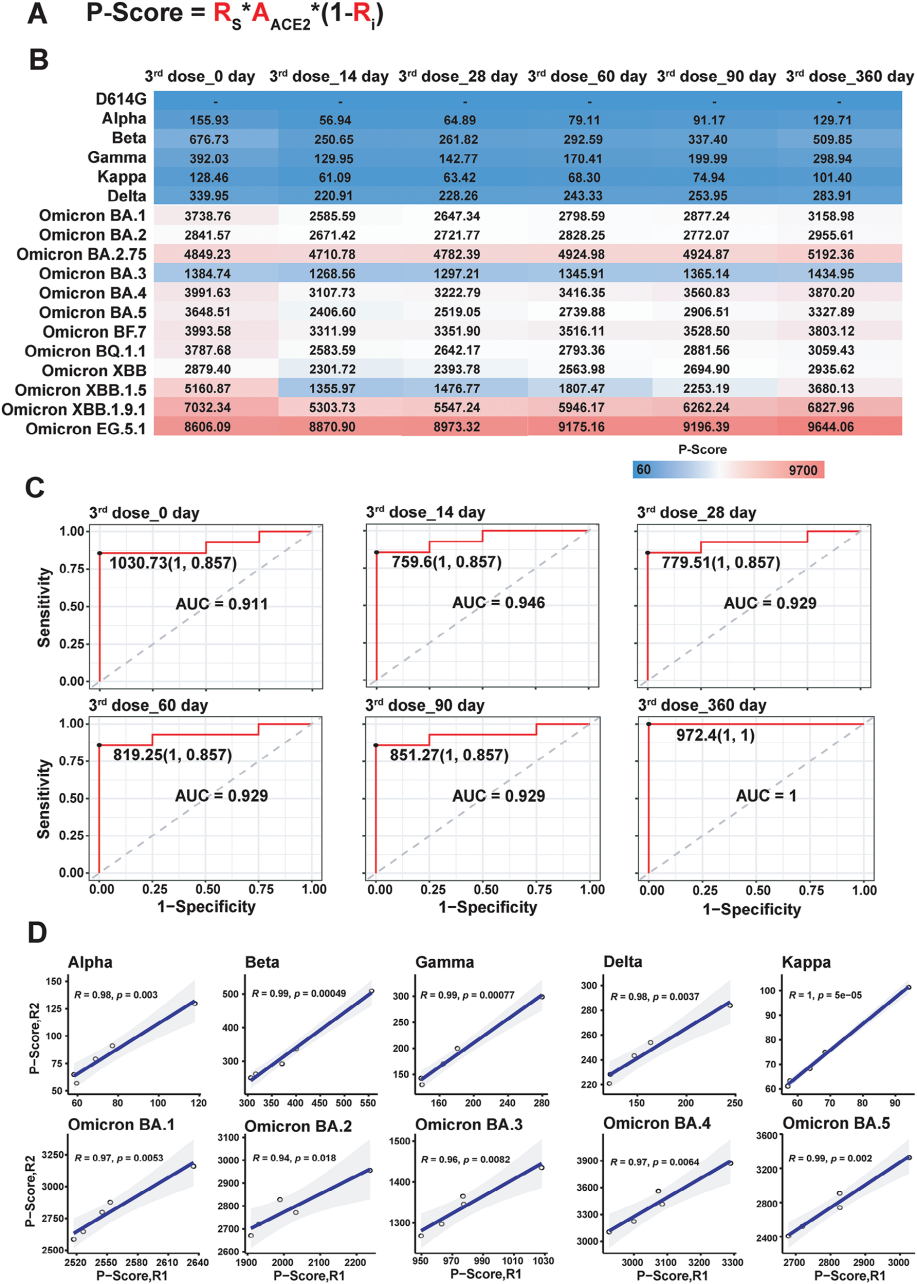
Development of a *P*‐Score for predicting the prevalence of SARS‐CoV‐2 variants. A) Describes the mathematical model for predicting the prevalence of SARS‐CoV‐2 variants in the population, including the *P*‐Score calculated using Spike‐ACE2 receptor binding affinity (*A*_ACE2_), the percentage of amino acids substitution in the RBD domain (*R*_S_), and the inhibition rate (*R*_I_, %) representing humoral protective immunity. B) Shows the prevalence score calculated for different SARS‐CoV‐2 variants in the population with the third doses of inactivated or recombinant RBD vaccine administration. C) Presents the accuracy of predicting SARS‐CoV‐2 Omicron variants in the inactivated or recombinant RBD vaccine administration population using *P*‐Score. The AUCs were obtained using the pROC package (v 1.18.2) and the optimal outpoints were selected with the Youden index which was also implemented in the coords function in the pROC package. D) The reproducibility of *P*‐Score predictions in two experiments conducted at different times within the same cohort.

Considering our findings, we developed a mathematical model aimed at predicting the transmission and prevalence of SARS‐CoV‐2 variants within populations. The *P*‐Score is derived by integrating the RBD mutation rate, ACE2 receptor binding, and proteomic data related to immune evasion (**Figure** **5**A). The P‐Score we conducted in this study to estimate prevalence of variants represented by the formula: *P*‐Score = *R*_S_*A_ACE2_*(1‐*R*_I_)*100%. Where, *R*_S_ represents the AA substitution mutation rate of the RBD region of the spike protein of SARS‐CoV‐2 variant, *A*_ACE2_ represents the spike protein‐ACE2 binding affinity, and *R*_I_ represents the inhibition rate (%). All three aspects are reported broadly to play key effects in the spread of SARS‐CoV‐2.^[^ ^46^ ^]^

In order to evaluate the performance of P‐Score in indicating the prevalence of SARS‐CoV‐2 variants, we collected the variants of concern (*V*OCs) or variants of interest (VOIs) from Nextrain (https://nextstrain.org/) and National Genomics Data Center (https://ngdc.cncb.ac.cn/ncov/monitoring/country/China) as the gold standard dataset (Table S5, Supporting Information). Based on the P‐Score of variants, we can predict whether the variants are VOC/VOIs or not through using a cutoff which optimized on the corresponding Receiver Operating Characteristic (ROC) curve. According to the gold standard and the prediction results of the P‐Score algorithm, a confusion matrix that depicts all four possible outcomes: true positive (TP), true negative (TN), false positive (FP), and false negative (FN) was summarized. And then the sensitivity and specificity can be calculated based on the two formulas: Sensitivity = TP/(TP + FN), Specificity = TN/(TN + FP).

The mutation rate within the RBD domain is sourced from the publicly available GISAID database (Figure S6, Supporting Information). The spike protein of emerging variants can be promptly obtained through mutagenesis and protein expression and purification. ACE2 receptor binding and population immunity information are accessible via our high‐throughput proteomic platform, with periodic updates based on vaccination and/or infection statuses. Diverging from earlier algorithmic models,^[^ ^24^, ^25^, ^26^, ^28^ ^]^ our model is firmly anchored in experimental data, facilitating real‐world predictions of SARS‐CoV‐2 variant prevalence ahead of large‐scale transmission.

To showcase our model's effectiveness, we applied it to a cohort that received a third‐dose inactivated or recombinant RBD vaccine administration. We determined serum NAbs to eighteen SARS‐CoV‐2 variants (D614G, Alpha, Beta, Gamma, Delta, Kappa, BA.1, BA.2, BA.3, BA.4, BA.5, BF.7, BA.2.75, BQ.1.1, XBB, XBB.1.5, XBB.1.9.1, and EG.5.1) before and at six‐time points (day 0, 14, 28, 60, 90, and 360) after vaccination (Figure S7, Supporting Information). Subsequently, we calculated the P‐Score for these variants within the population that received the third‐dose vaccination. The results indicated that the *P*‐score of non‐Omicron variants was low, ranging from 56.94 to 679.73. The P‐Score decreased from day 0 to day 90, with a slight increase at day 360. In contrast, the *P*‐Score was significantly higher for Omicron variants, ranging from 1268.56 to 9644.06. The observed change in P‐Score for the majority of Omicron variants mirrored that of non‐Omicron variants, suggesting potential protection conferred by inactivated or recombinant RBD vaccine administration. These findings imply that the transmission risk in the vaccinated population might increase with time after vaccination due to waning protective immunity. Notably, the P‐Score of EG.5.1 was unaffected by vaccination and continually increased with time, possibly explaining its prevalence in this year.^[^ ^47^ ^]^ The data yielded AUCs of 0.91‐1.00 for predicting the prevalence of Omicron variants in the vaccinated population with the threshold of 759.6‐1030.73 according to the GISAID database.^[^ ^1^ ^]^ Comparatively, the predictive performance of P‐Score obtained with immunity data, with and without ACE2 binding, was 0.889–0.946 and 0.75–0.929, respectively (Figure S8, Supporting Information), underscoring the necessity of predicting new variant prevalence using the RBD mutation rate, ACE2 receptor binding, and population immunity.

Lastly, we scrutinized the reproducibility of *P*‐Score predictions in two experiments conducted at different times within the same cohort. The results showed that the Pearson correlations of P‐Score calculations ranged from 0.94 to 1.00 for non‐Omicron variants and Omicron variants (BA.1–BA.5) (Figure 5D). Collectively, these findings validate the reliability of our model in predicting the potential prevalence of new variants. Nevertheless, it is crucial to note that the transmission and prevalence of SARS‐CoV‐2 variants are also influenced by factors such as vaccination type, doses, timing, as well as accessibility, and individual immunity.^[^ ^24^ ^]^

### 2.7 Comprehensive Profiling of Mouse Immune Responses to SARS‐CoV‐2 Variants Induced by Wild‐Type (WT), Delta, and Omicron BA.1 mRNA Vaccination

**Figure 6:**
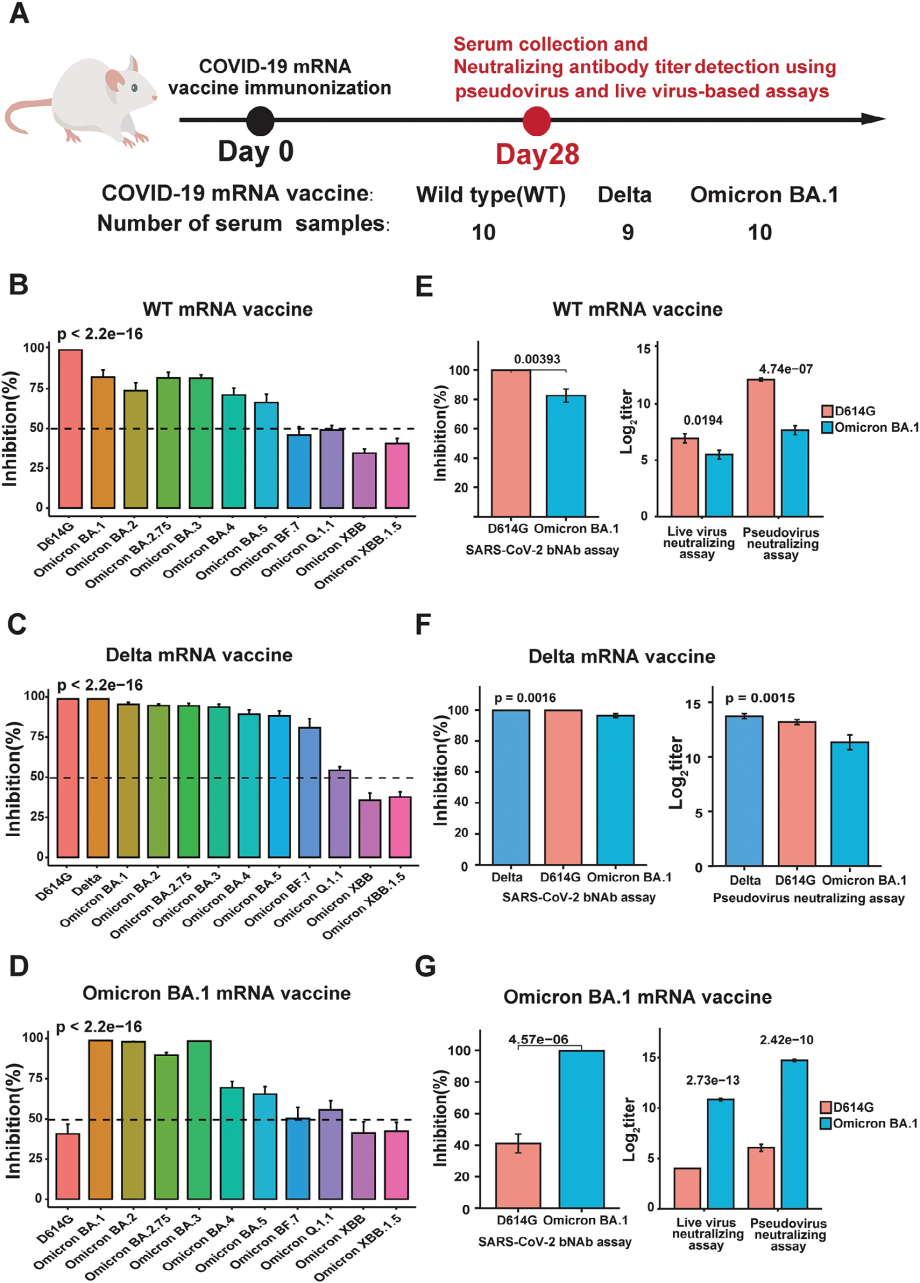
Detection of serum NAbs in mouse model vaccinated with WT, Delta, and BA.1 mRNA vaccination. A) Illustrates the timeline of serum sample collection in vaccinated mice. 10 mice were vaccinated with WT vaccine, 9 mice were vaccinated with Delta vaccine, and 10 mice were vaccinated with BA.1 vaccine. The serum was collected 28 days after vaccination. B–D) Shows the inhibition rate (%) of NAbs against different SARS‐CoV‐2 variants in mice vaccinated with WT, Delta, and BA.1 mRNA vaccines, respectively. The comparison of the mean of the inhibition rate (%) of NAbs against different SARS‐CoV‐2 variants was conducted using ANOVA test. E–G) Validates proteomic results using pseudovirus and live virus‐based neutralization assays. The comparison of the mean of serum NAb inhibition rates or titers in mice vaccinated by different vaccines was conducted using Welch's t‐test or Wilcoxon rank‐sum test.

To explore the vaccination‐induced immunity conferred by distinct vaccines, we analyzed serum samples from a mouse model (*n* = 29), collected 28 days post‐vaccination with mRNA vaccines targeting WT, Delta, and Omicron BA.1 variants, as outlined in **Figure** **6**A.^[9,^ ^48^ ^]^ We profiled the serum NAbs against ten Omicron variants (Omicron BA.1, BA.2, BA.3, BA.4, BA.5, BF.7, BA.2.75, BQ.1.1, XBB, and XBB.1.5), using D614G as the reference. The inhibition rate (%) was calculated without adjusting for background, given the unavailability of pre‐vaccination serum samples.

In Figure 6B, the data illustrates that serum NAbs induced by the WT vaccine exhibited the highest inhibition rate (%) against D614G, followed by BA.1, BA.2.75, BA.3, BA.4, BA.5, BQ.1.1, BF.7, with the XBB variants (XBB and XBB.1.5) displaying the lowest inhibition (Figure 6B). A similar pattern was observed with the Delta vaccine, where serum NAbs demonstrated the highest inhibition rate (%) against Delta and D614G, followed by BA.1, BA.2.75, BA.3, BA.2, BA.4, BA.5, BF.7, BQ.1.1, XBB.1.5, and XBB (Figure 6C). In contrast, distinct profiles emerged for the Omicron BA.1 vaccine, with serum NAbs exhibiting the highest inhibition rate (%) against BA.1, followed by BA.3, BA.2, and BA.2.75. The NAb inhibition rate (%) significantly decreased for BA.4, BA.5, BQ.1.1, BF.7, with the XBB variants (XBB and XBB.1.5) and D614G showing the lowest inhibition (Figure 6D).

These findings were selectively validated through pseudovirus and live virus‐based neutralization assays, wherein NAbs induced by WT, Delta, and BA.1 vaccines displayed the highest inhibition against WT, Delta, and BA.1 variants, respectively (Figure 6E–G). The results align with pseudovirus and live virus‐based neutralization assays and previously reported findings.^[^ ^49^, ^50^, ^51^ ^]^

### 2.8 Dynamic Evolution of Omicron Variants' Immunological Classification under the Influence of Different SARS‐CoV‐2 Variant Vaccines

**Figure 7:**
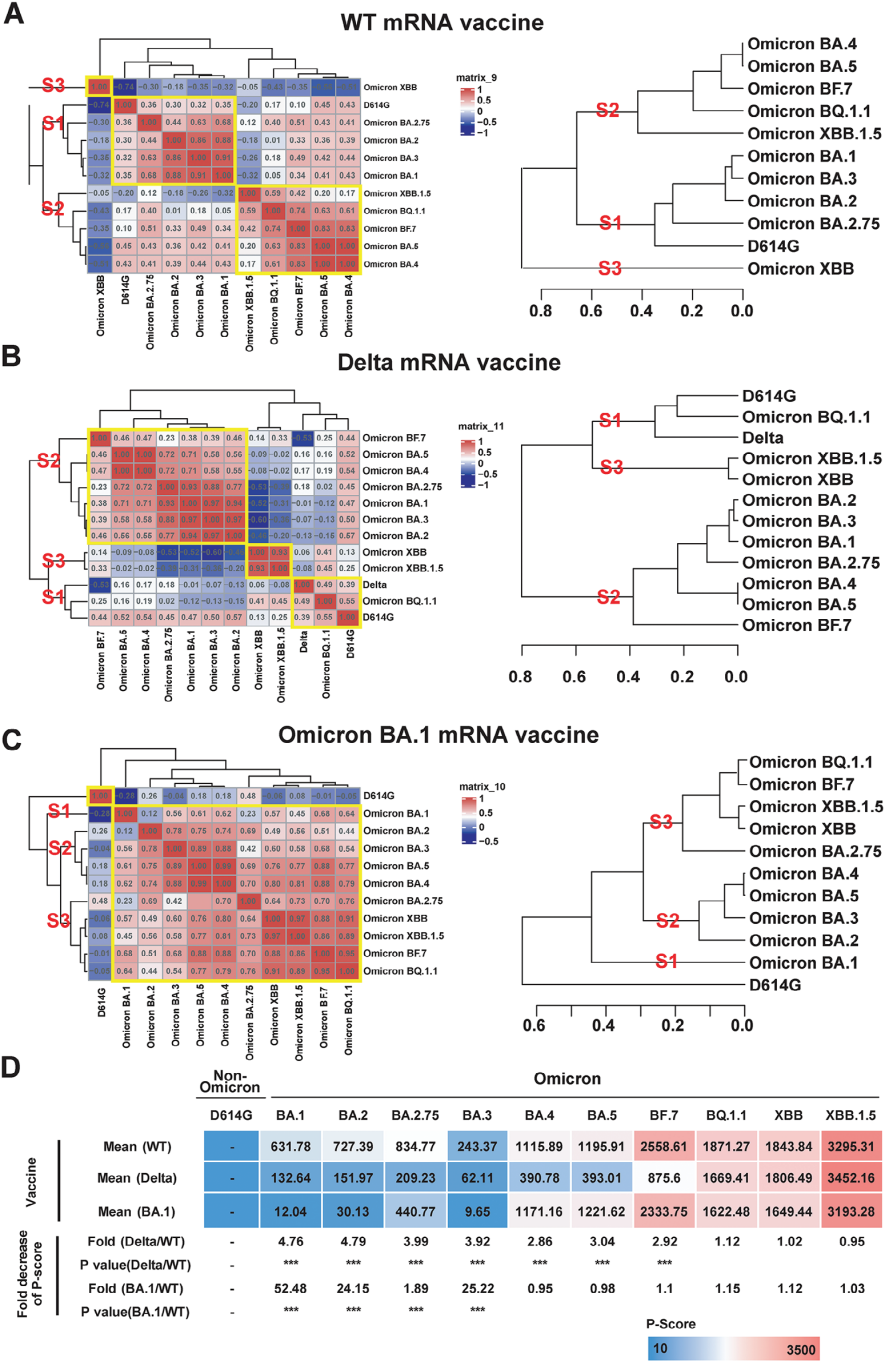
Classification of vaccination immunity to Omicron variants in mice received WT, Delta, and BA.1 vaccines. A–C) Compares human protective immunity of SARS‐CoV‐2 non‐Omicron and Omicron variants in mice with WT, Delta, and BA.1 vaccination, respectively. The nonbiased hierarchical clustering was executed and the heat map was visualized using Pearson's correlation distance/complete linkage method implemented in the R package Complexheatmap (version 2.16.0). D) Displays the prevalence score for SARS‐CoV‐2 variants in mice vaccinated with WT, Delta, and BA.1 mRNA vaccines. The decreased folds and *p* values of prevalence score of different SARS‐CoV‐2 variants in mice vaccinated with Delta and BA.1 mRNA vaccines were calculated compared to WT vaccine. The p values were calculated using Welch's t‐test or Wilcoxon rank‐sum test, in which the asterisks (^***^) represent the *p* < 0.001.

To unravel the intricate evolution of Omicron subtypes influenced by diverse vaccines, we employed unbiased hierarchical and correlation analyses of NAb responses to D614G and Omicron variants, respectively (**Figure** **7**). In Figure 7A, despite D614G clustering with variants in Omicron Sub‐cluster 1 (BA.1, BA.2, BA.3, and BA.2.75), the correlations were notably low (0.33 to 0.35), consistent with results from the inactivated or recombinant RBD vaccine administration cohort (Figure 3D). In Sub‐cluster 2, BA.4, BA.5, BQ.1.1, BF.7, and XBB.1.5 were grouped, while XBB formed Sub‐cluster 3.

For the Delta vaccine, BQ.1.1 clustered in Sub‐cluster 1 (Figure 7B), with BA.1, BA.2, BA.3, BA.2.75, BA.4, BA.5, and BF.7 in Sub‐cluster 2, and XBB and XBB.1.5 in Sub‐cluster 3. Regarding the BA.1 vaccine, BA.1 formed Sub‐cluster 1, BA.2‐BA.5 constituted Sub‐cluster 2, and BQ.1.1, BF.7, XBB, and XBB.1.5 clustered as Sub‐cluster 3 (Figure 7C). These results underscore that vaccine‐induced immune responses reshape the classification of responses to SARS‐CoV‐2 variants, and Omicron variants continue to evolve into distinct subtypes based on vaccination.

The function of immunological classification can be indicated in the vaccination immunity against the prevalence of different Omicron sub‐lineage variants. Utilizing the *P*‐Score in a mouse model, we analyzed the shifts in Omicron variant prevalence regulated by different mRNA vaccines (Figure 7D). Results for the WT vaccine indicated a relatively low prevalence risk for BA.1, BA.2, BA.3, and BA.2.75, with P‐Scores ranging from 243.37 to 834.77. However, the *P*‐Score significantly increased for BA.4, BA.5, BF.7, BQ.1.1, XBB, and XBB.1.5.

Concerning the Delta vaccine, a relatively low prevalence risk was noted for BA.1, BA.2, BA.3, BA.2.75, BA.4, BA.5, and BF.7, with *P*‐Scores ranging from 62.11 to 875.60. However, the *P*‐Score significantly increased for BQ.1.1, XBB, and XBB.1.5, with *P*‐Scores ranging from 1669.41 to 3452.16.

The BA.1 vaccine displayed a relatively low prevalence risk for BA.1, BA.2, BA.3, and BA.2.75, with *P*‐Scores from 9.65 to 440.77. However, the *P*‐Score significantly increased for BA.4, BA.5, BF.7, BQ.1.1, XBB, and XBB.1.5, with *P*‐Scores from 1171.16 to 3193.28. These findings suggest that these three vaccines may not offer sufficient protection against BQ.1.1, XBB, and XBB.1.5 variants in a mouse model. However, it is crucial to note that the species difference between humans and mice may lead to variations in serum NAb and *P*‐Score determination.

### 2.9 Impact of BA.4/5 mRNA Vaccination on Omicron Sub‐Lineage Variant Prevalence in Individuals

**Figure 8:**
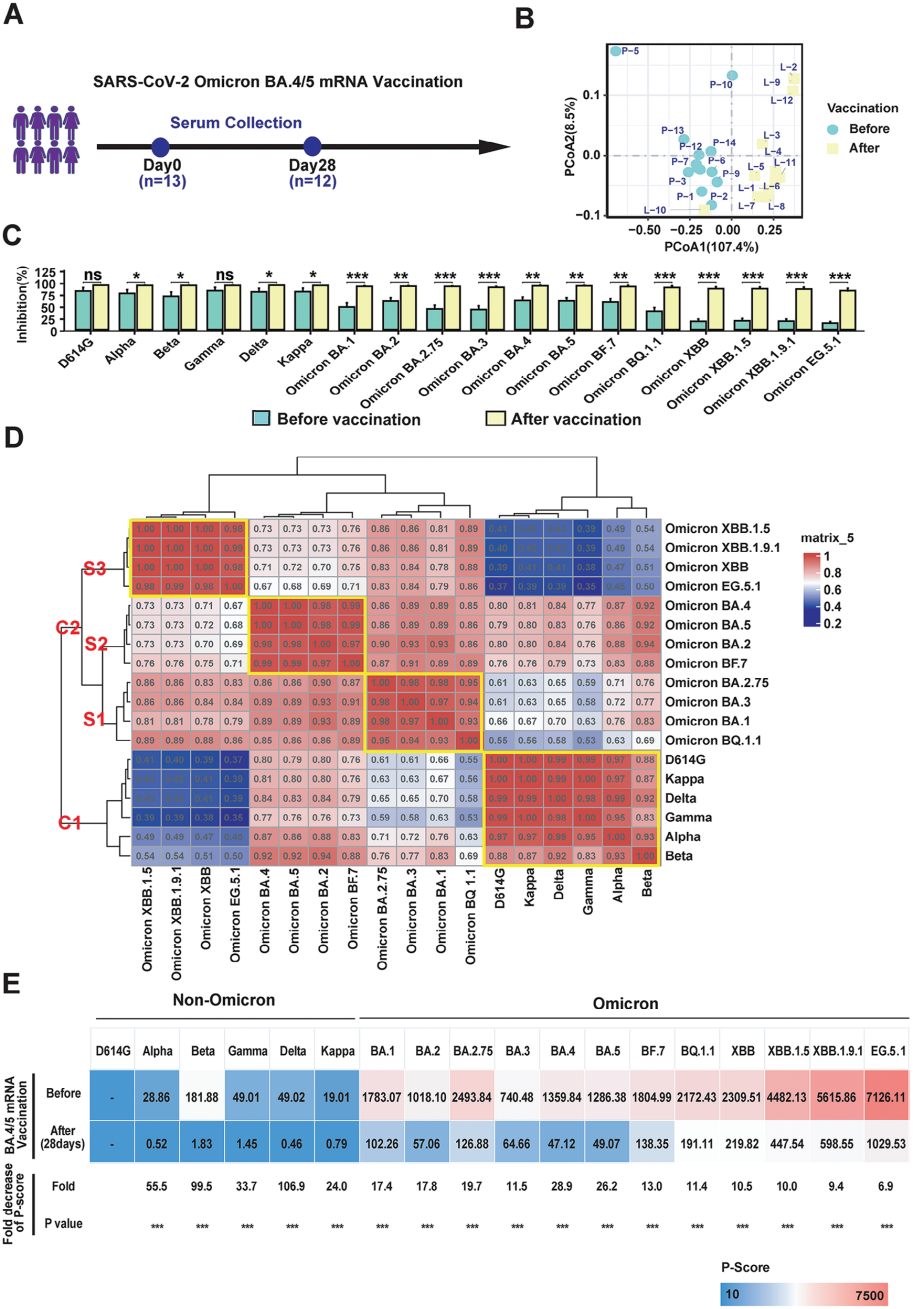
Detection of serum NAbs in individuals with BA.4/5 mRNA vaccination. A) Illustrates the timeline of serum samples collected before (*n* = 13) and after (*n* = 12) vaccination with the BA.4/5 mRNA vaccine. B) Conducts principle component analysis of individuals before and after vaccination. C) Shows the inhibition rate (%) of NAbs against different SARS‐CoV‐2 variants in individuals before and after vaccination, respectively. The *p* values were calculated utilizing Welch's *t*‐test or Wilcoxon rank‐sum test. ns *p* > 0.05; ^*^ *p* < 0.05; ^**^ *p* < 0.01; ^***^ *p*  < 0.001. D) Executes non‐biased hierarchical clustering analysis of vaccination immunity and SARS‐CoV‐2 non‐Omicron and Omicron variants. The nonbiased hierarchical clustering analysis was executed by the R package Complexheatmap (version 2.16.0). E) Displays the P‐Score for SARS‐CoV‐2 variants in individuals vaccinated with BA.4/5 mRNA vaccine. The decreased folds and *p* values of prevalence score of different SARS‐CoV‐2 variants in individuals before and after vaccination. The *p* values were calculated using Welch's *t*‐test or Wilcoxon rank‐sum test, in which the asterisks (^***^) represent the *p* < 0.001.

To discern the influence of BA.4/5 mRNA vaccination on protective immunity and the potential prevalence of SARS‐CoV‐2 variants, we collected serum samples before (*n* = 13) and after (*n* = 12) vaccination with the BA.4/5 mRNA vaccine (**Figure** **8**A). The inhibition rate (%) of serum NAbs against eighteen SARS‐CoV‐2 variants was determined using the SARS‐CoV‐2 bNAb assay, visualized through principal component analysis (Figure 8B). The results showcased a clear separation between serum samples collected before and after vaccination, signifying the activation of protective immunity.

The inhibition rate (%) to SARS‐CoV‐2 variants is presented in Figure 8C. Before BA.4/5 vaccination, serum NAbs exhibited the strongest reaction to non‐Omicron variants (Gamma, D614G, Kappa, Delta, Alpha, and Beta variants), significantly decreasing against Omicron variants (BA.4, BA.5, BA.2, BF.7, BA.1, BA.2.75, BA.3, BQ.1.1, XBB.1.5, XBB, XBB.1.9.1, and EG.5.1). The results suggested that these individuals might be vaccinated with non‐Omicron vaccine before. However, the inhibition rate (%) to these Omicron variants significantly increased post‐vaccination, indicating the effectiveness of the BA.4/5 mRNA vaccine.

We conducted an unbiased hierarchical analysis of eighteen variants based on serum NAbs (Figure 8D). In alignment with our earlier findings (Figure 3D), all non‐Omicron variants clustered together as the non‐Omicron type. In comparison, Omicron variants were clustered into three subtypes: BA.1, BA.3, BA.2.75, BQ.1.1 formed Sub‐cluster 1; BA.2, BA.4, BA.5, BF.7 comprised Sub‐cluster 2; XBB.1.5, XBB, XBB.1.9.1, and EG.5.1 clustered as Sub‐cluster 3. These results further underscore the evolution of immunological classification in response to vaccination immunity.

We calculated P‐Scores for all variants in individuals vaccinated with the BA.4/5 mRNA vaccine (Figure 8E). Before vaccination, *P*‐Scores ranged from 19.01 to 181.88 for non‐Omicron variants (Gamma, D614G, Kappa, Delta, Alpha, and Beta variants). However, *P*‐Scores were significantly higher for Omicron variants (BA.4, BA.5, BA.2, BF.7, BA.1, BA.2.75, BA.3, BQ.1.1, XBB.1.5, XBB, and XBB.1.9.1), with EG.5.1 registering the highest. Post‐vaccination, the potential prevalence risk significantly decreased for Omicron variants (BA.4, BA.5, BA.2, BF.7, BA.1, BA.2.75, BA.3, BQ.1.1, XBB.1.5, XBB, and XBB.1.9.1), with fold changes of 9.4–28.9‐folds. However, the prevalence risk decreased by 6.9‐folds for the EG.5.1 variant, with a P‐Score exceeding 1000, indicating EG.5.1 may still pose a potential risk for viral transmission.

**Figure 9:**
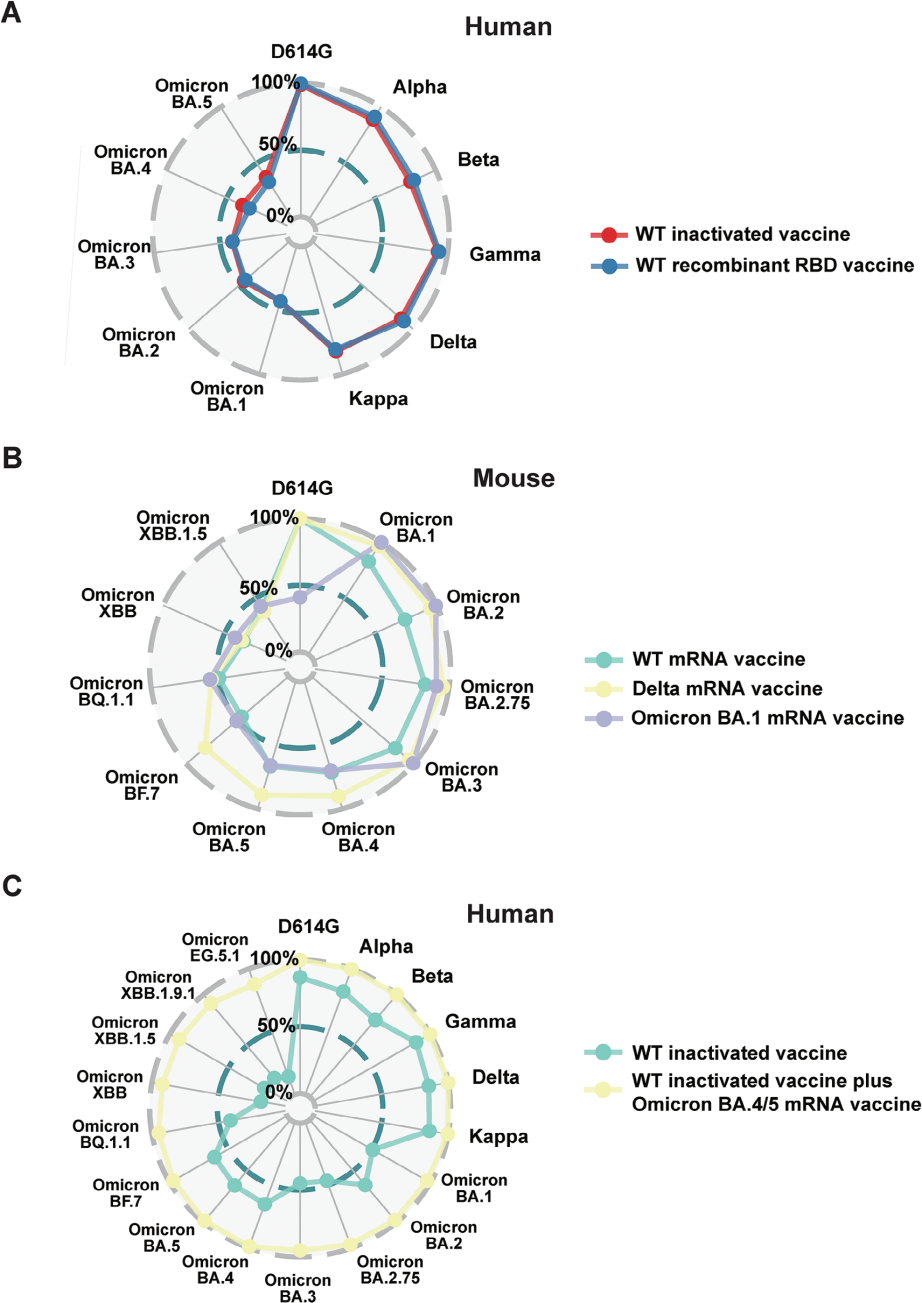
Comparison of the inhibition rate (%) of the serum NAbs in human and mouse cohorts vaccinated by different SAR‐CoV‐2 variants. A) The radar plot describes the differences in the inhibition rates (%) of serum NAbs against various SARS‐CoV‐2 variants in the human cohort vaccinated with the WT inactivated vaccine and the WT recombinant RBD vaccine. B) The radar plot describes the differences in the inhibition rate (%) of serum NAbs against various SARS‐CoV‐2 variants in the mouse cohort vaccinated with WT, Delta, and Omicron BA.1 mRNA vaccine. C) The radar plot describes the difference in the inhibition rate (%) of serum NAbs against various SARS‐CoV‐2 variants in the human cohort before and after Omicron BA.4/5 mRNA vaccine administration.

Finally, we summarized the broad neutralizing activity of various vaccines in **Figure** **9** and Table S6 (Supporting Information). The results indicated that the WT vaccines—including the inactivated vaccine, recombinant RBD vaccine, and mRNA vaccine—provide protection against SARS‐CoV‐2, specifically against non‐Omicron variants (D614G, Beta, Gamma, Delta, and Kappa). Conversely, vaccines derived from the Delta and Omicron BA.1 variants extend their neutralizing activity to cover the Omicron BA.1 – BF.7 variants but are less effective against the subsequently evolved Omicron variants, namely BQ.1, XBB, and XBB.1.5 (Figure 9B). Notably, the combination of the WT‐inactivated vaccine and the Omicron BA.4/5 mRNA vaccine exhibited broad neutralizing activity against all SARS‐CoV‐2 variants detected in this study (Figure 9C). These findings suggest a viable approach to achieving broad neutralization activity through hybrid vaccination involving vaccines derived from different SARS‐CoV‐2 serotypes (Figures 3D and 8D).

## 3 Discussion

Understanding the synergistic evolution of SARS‐CoV‐2 and vaccination immunity is crucial for assessing population immunity in reality scenarios, developing vaccines for emerging variants, and formulating effective epidemic prevention and control strategies.^[^ ^2^, ^52^ ^]^ In contrast to prior algorithm‐based models for predicting new variants with immune evasion,^[^ ^9^, ^10^, ^11^, ^12^, ^13^, ^14^ ^]^ our proteomics platform enables the direct acquisition of ACE2 receptor binding and population immunity. It facilitates the forecasting of new variants, taking into account vaccination immunity within specific regions.

In this study, three key achievements were realized through our proteomic platform. First, we observed that the efficacy of vaccination immunity against SARS‐CoV‐2 variants is associated with vaccine type, vaccination doses, and the time duration post‐vaccination (Figure 2). The co‐evolution of SARS‐CoV‐2 genetic mutations and vaccination immunity was systematically analyzed in humans and mice administered with the WT inactivated vaccine, as well as WT, Delta, BA.1, and BA.4/5 mRNA vaccines, respectively. Results revealed that serum NAbs induced by the WT inactivated vaccination and WT and Delta mRNA vaccinations exhibit greater specificity toward non‐Omicron variants compared to Omicron variants (Figures 3, 6 and 6C). Conversely, vaccination immunity induced by BA.1 and BA.4/5 mRNA vaccines demonstrates a higher specificity for Omicron variants than non‐Omicron variants (Figures 6D and 8C), particularly for the latest XBB sub‐lineages (XBB, XBB.1.5, XBB.1.9.1, and EG.5.1).

In addition, we observed that the inhibition rate (%) of NAbs can be strengthened by the vaccination doses, in which the inhibition of NAbs to different SARS‐CoV‐2 variants was significantly increased with the third‐dose vaccination (Figure 3C). The results were according to reported studies on the inactivated and mRNA vaccines.^[^ ^53^, ^54^, ^55^ ^]^ It was found that the memory B cell clones with significantly higher levels of somatic mutations were increased by boosted vaccination.^[^ ^56^, ^57^ ^]^ This result may increase the diversity of NAbs and improve the breadth and cross‐reactivity of NAb response to different SARS‐CoV‐2 variants.^[^ ^53^, ^54^, ^55^ ^]^ In addition, the glycosylation of neutralizing antibodies was also found to be changed with the vaccination, and the transient increase of galactosylation, sialylation, fucosylation of anti‐S IgG1 antibody was observed by Liquid chromatography‐Mass spectrometry (LC‐MS). However, the underlying mechanism is unclear.^[^ ^58^, ^59^ ^]^

Second, we address a longstanding question raised by Simon‐Loriere and Schwartz in 2022 regarding the classification of humoral responses to SARS‐CoV‐2 variants during its evolution.^[^ ^2^ ^,^ ^30^ ^,^ ^31^ ^,^ ^33^, ^38^ ^]^ Our study not only confirms Simon–Loriere's hypothesis but also provides direct evidence supporting the classification of vaccination immunity (Figures 3, 7, and 8D). Furthermore, we demonstrate that the Omicron type adapts to genetic evolution, a phenomenon regulated by vaccination and native infection. For instance, the serotype of Omicron variants' immunity was classified into two subtypes (BA.1 as Sub‐cluster 1 and BA.2‐5 as Sub‐cluster 2) by the WT inactivated or recombinant RBD vaccine administration (Figure 3D). WT mRNA vaccine classified them into three subtypes (BA.1‐3 and BA.2.75 as Sub‐cluster 1; BA.4, BA.5, BF.7, BQ.1.1, and XBB.1.5 as Sub‐cluster 2; XBB as Sub‐cluster 3) (Figure 7A). Delta mRNA vaccine categorized them into three subtypes (BA.1‐5, BF.7, BA.2.75 as Sub‐cluster 2; XBB and XBB.1.5 as Sub‐cluster 3; BQ1.1 as Sub‐cluster 1) (Figure 7C). BA.1 mRNA vaccine classified three subtypes (BA.1 as Sub‐cluster 1; BA.2‐5 as Sub‐cluster 2; BA2.75, BF.7, BQ.1.1, XBB, and XBB.1.5 as Sub‐cluster 3) (Figure 7E). BA.4/5 mRNA vaccine identified three subtypes (BA.1, BA.3, BA.2.75, and BQ.1. as Sub‐cluster 1; BA.2, BA.4, BA.5, and BF.7 as Sub‐cluster 2; XBB, XBB.1.5, XBB.1.9.1, and EG.5.1 as Sub‐cluster 3) (Figure 8D).

Third, while significant progress has been made in predicting new variants using various algorithms,^[^ ^24^, ^25^, ^26^, ^27^, ^28^, ^29^ ^]^ the accuracy of prediction models remains insufficient to cover all mutations generated by natural evolution.^[^ ^26^, ^28^ ^]^ Moreover, beyond genetic mutations, the prevalence of new variants depends on ACE2 binding affinity and population immunity resulting from vaccination and natural infection. Traditional methods with low throughput, such as pseudovirus and live virus‐based neutralization assays, limit the acquisition of a large amount of immunity data from the population. Addressing this challenge, we developed a high throughput proteomic assay, enabling the rapid acquisition of ACE2 binding affinity and immune evasion information in populations exposed to SARS‐CoV‐2 variants.^[^ ^34^ ^]^ Furthermore, we introduced a mathematical model, P‐Score, incorporating RBD mutation rate, ACE2 receptor binding, and immune evasion. This model demonstrated high accuracy (AUC: 0.911‐1.00) in the group of individuals received with the third dose inactivated or recombinant RBD vaccine administration (Figure 5C; Figure S8, Supporting Information). Besides, we also validated the P‐Score algorithm though applying it to evaluate the vaccine effectiveness in two different cohorts which vaccinated by different mRNA vaccines (Figures 7D and 8E).

Using P‐Score, we highlight the high prevalence risk of Omicron variants for hosts vaccinated with inactivated or recombinant RBD vaccine (Figure 5) and different mRNA vaccines (Figures 7D and 8E). Additionally, we show that the prevalence risk of Omicron variants can be significantly reduced by the hybrid vaccination of WT inactivated vaccine and BA.4/5 mRNA vaccines (Figures 8E and 9). Notably, the *P*‐Score indicates that BA.4/5 mRNA vaccination can significantly decrease the prevalence risk of Omicron sub‐lineage variants from 6.9 to 28.9‐folds. However, the P‐Score of EG.5.1 variant (1029.53) approaches the cut‐off (759.6–1030.73 in Figure 4C), implying a potential risk associated with EG.5.1 variant (Figure 8E). These findings suggest that the timely update of spike antigen in mRNA vaccines, in accordance with the co‐evolution of SARS‐CoV‐2 genetic and vaccination immunity, may be an effective approach to protect the population against viral infection and prevalence.

Several limitations are remained in this study. First, the relationship between the inhibition rate (%) obtained by the proteomic assay and SARS‐CoV‐2 infection rate remains unclear. To address this question, we compared our assay with the IgG serological assay, the FDA‐approved cPass sVNT assay, pseudovirus‐based neutralization assay and live virus‐based neutralization assay.^[^ ^34^ ^]^ The result showed that our bNAb neutralizing assay was significantly correlated with the IgG serological assay (*R* = 0.89), the FDA‐approved cPass sVNT assay (*R* = 0.93), pseudovirus‐based neutralization assay (*R* = 0.96 for D614G, *R* = 0.66 for BA.1) and live virus‐based neutralization assay (*R* = 0.79 for D614G, *R* = 0.64 for BA.1),^[^ ^34^ ^]^ indicating the association between our surrogate neutralizing with the humoral protective immunity.^[^ ^60^ ^]^

Second, the SARS‐CoV‐2 WT trimer protein was not utilized due to its unavailability from vendors during the experimental phase. This could potentially be attributed to the instability of the SARS‐CoV‐2 S protein in its metastable prefusion state, which may hinder its purification and affect its immunogenic properties.^[^ ^61^, ^62^ ^]^ In addition, according to previous studies, the characteristics of the new variant against the ancestral form were compared in human cells and animal models.^[^ ^63^, ^64^ ^]^ The results showed that the D614G variant exhibits more efficient infection, replication, and competitive fitness in primary human airway epithelial cells but maintains similar morphology and in vitro neutralization properties, compared with the ancestral WT virus.

Third, the number of samples employed in this work was limited. It would be significant to expand our study to a large healthy and immunosuppressive (i.e., HIV patients) population to study the adaptive evolution of population immunity through various vaccines (e.g., mRNA) and/or native infection.

Finally, the prediction model should undergo periodic iteration with the expansion of the SARS‐CoV‐2 genome database and population immunity database, along with the development of prediction algorithms.^[^ ^26^, ^28^ ^]^ These efforts will be instrumental in achieving active immunity by designing more effective COVID‐19 vaccines, tailoring vaccination strategies to target populations, develop more effective therapeutic treatments for immunosuppressive patients and enhancing public health management.

## 4 Conclusion

In this study, we systematically elucidated the co‐evolution of SARS‐CoV‐2 genetic mutation, ACE2 receptor binding and vaccination immunity, and classification of protective immunity by vaccines and formulated a prediction model based on population immunity to assess the prevalence risk of new SARS‐CoV‐2 variants predicted by algorithms or newly emerged before widespread transmission. Our findings significantly contribute to comprehending population immunity variations resulting from diverse vaccination methods and natural infections. This, in turn, can provide valuable insights for vaccine development and guide public health strategies, particularly when integrated with machine learning tools in future endeavors.

## 5 Experimental Section

### Collection of Serum Samples

In this study, a total of 768 clinical serum samples were systematically collected. These samples were procured from both the Beijing Ditan Hospital and Beijing Chaoyang Hospital, as detailed in Table S1 (Supporting Information). The assortment of serum samples included pre‐vaccination specimens, along with samples acquired at specific time points: 14 days after the initial vaccine dose, 28 days after the second vaccine dose, and at intervals of 0, 14, 28, 60, 90, and 360 days following the administration of the third vaccine dose.

The blood collection process involved obtaining whole blood in a vacutainer tube, followed by centrifugation at 4000×g at room temperature (RT) for 10 min to facilitate serum separation. Subsequently, the isolated serum was carefully transferred to clean tubes and meticulously stored at −80 °C until required for analysis. It is noteworthy that our research protocol received approval from the Ethics Committee (No. 2021‐010‐01), and informed consent exemption was obtained in advance of serum collection.^[^ ^67^ ^]^

### Detection of Serum NAbs Using SARS‐CoV‐2 bNAb Assay

The SARS‐CoV‐2 bNAb assay kit was developed by ProteomicsEra Medical Co., Ltd (Beijing, China) as previously described.^[^ ^34^ ^]^ To initiate the assay, a 96‐well plate was prepared, and each well was supplemented with a 50 µL mixed solution containing 12 distinct SARS‐CoV‐2 spike trimer protein‐coupled beads, with each type comprising 2500 beads. Simultaneously, 50 µL of serum samples, meticulously diluted to a ratio of 1:20 with PBST‐B, were added to each well. Following a 2 h incubation period at room temperature on a shaker, the beads underwent a thorough wash cycle with PBST‐B repeated three times. Subsequently, the beads were subjected to another incubation step, this time with 50 µL of biotinylated ACE2 (0.5 µg mL^−1^), for an additional hour at room temperature. Post‐incubation, the binding interaction between ACE2 and spike trimer proteins was probed by introducing 50 µL of SA‐PE (2 µg mL^−1^), and the amalgamation was allowed to incubate for 30 min at room temperature. Following a final washing step with PBST‐B performed twice, the beads were resuspended in a 200 µL PBST‐B solution. For signal detection, the fluorescent output was measured at 200 beads/region utilizing the EasyCell flow cytometry system (Wellgrow Technology Co., Ltd., Beijng, China), employing excitation wavelengths of 532 and 635 nm.

### Detection of NAbs Using Pseudovirus‐Based Neutralization Assay

Huh7 cells (JCRB, 0403) were meticulously seeded in 96‐well plates at a density of 20000 cells per well and incubated for a duration of 20–28 h to ensure optimal cell adherence and growth. Serum samples, subjected to serial three‐fold dilutions commencing at 1:10, were then incubated with 650 TCID50 of the pseudovirus for a precisely controlled duration of 1 h at 37 °C. As a crucial negative control, Dulbecco's Modified Eagle Medium (DMEM) was employed. Following the incubation period, the supernatant was carefully aspirated, making way for the addition of luciferase substrate to each well. A subsequent 2 min incubation in darkness at room temperature facilitated optimal enzymatic reactions. Luciferase activity, indicative of NAb presence, was quantified using the GloMax® 9633 Microplate Luminometer (Promega, Madison, USA).

### Detection of NAbs Using Live Virus‐Based Neutralization Assay

To assess the SARS‐CoV‐2‐specific NAb titer in serum, a cytopathic effect (CPE)‐based microneutralization assay was employed, utilizing the SARS‐CoV‐2 virus strain BetaCoV/Beijing/IME‐BJ01/2020 (Accession No. GWHACAX01000000) and Vero cells (ATCC, CCL81). Serum samples underwent heat inactivation for 30 min at 56 °C and were subsequently two‐fold serially diluted (ranging from 1:4 to 1:2048) using Dulbecco's Modified Eagle Medium (DMEM) from Thermo Fisher Scientific. These dilutions were then mixed with an equivalent volume of the virus solution to achieve a 50% tissue culture infectious dose (TCID50) of 100 in each well.

The serum‐virus mixture underwent an incubation period of 1 h at 37 °C before being added to 96‐well plates containing semi‐confluent Vero cells with a density exceeding 80%. Following a subsequent 3‐day incubation at 37 °C, CPEs on Vero cells were meticulously observed using an inverted microscope. The neutralizing titer was determined as the reciprocal of the highest sample dilution that successfully protected a minimum of 50% of cells from CPE. In instances where no neutralization reaction was discernible at the initial serum dilution (1:4), an arbitrary titer of 2 (half of the limit of quantification) was reported.

### Data Analysis

In evaluating the inhibition rate (%) of serum NAbs against various SARS‐CoV‐2 variants, a meticulous data analysis pipeline was employed. The raw inhibition rates for each variant underwent a normalization process using minimum–maximum normalization, ensuring a standardized comparison. Subsequently, the pre‐processed data were subjected to comprehensive downstream analyses.

Statistical analyses were conducted utilizing ANOVA, Welch's *t*‐test or Wilcoxon rank‐sum test, with statistical significance set at a *p* value < 0.05. Pearson's correlation coefficients, calculated through the cor function, facilitated the exploration of correlations between distinct SARS‐CoV‐2 variants. For unbiased hierarchical clustering analysis, Pearson's correlation distance and the complete linkage method were implemented, leveraging the R package Complexheatmap (version 2.16.0).^[^ ^68^ ^]^

To unravel the dynamic interplay between SARS‐CoV‐2 and vaccination immunity, a homological analysis of SARS‐CoV‐2 variants was executed. This involved multi‐sequence alignments among their spike protein sequences, culminating in the construction of a phylogenetic tree. The R packages seqinr (v4.2‐30), msa (v1.32.0), and ape (v5.7‐1) were instrumental in facilitating this homological analysis.^[^ ^69^, ^70^ ^]^ Furthermore, the ROC analysis of the P‐Score was conducted using the R package pROC (v 1.18.2).^[^ ^71^ ^]^ All‐encompassing data analyses were carried out in R (v4.2.3) under the R Studio (v 19.1.3) environment, ensuring robust and reproducible outcomes in adherence to best practices in statistical analysis and bioinformatics.

The PCA was carried according to the following procedure^[^ ^72^ ^]^: 1) Data Standardization: The matrix of NAbs inhibition rate (%) was standardized to have a mean of 0 and a standard deviation of 1 for each feature. 2) Compute the Covariance Matrix: The covariance matrix of the standardized matrix was calculated. This matrix represents the relationship between the different features in the dataset. 3) Eigenvalue Decomposition: Eigenvalue decomposition was performed on the covariance matrix to obtain the eigenvectors and eigenvalues. 4) Rank the Eigenvectors: The eigenvectors were ranked in descending order based on their corresponding eigenvalues. The eigenvectors with the highest eigenvalues represent the principal components of the dataset. 5) Select the Principal Components: The top two eigenvectors were selected to form the new subspace, which corresponded to the desired number of dimensions for the reduced dataset.

### Molecular Dynamics Simulations

In order to investigate the binding characteristics of NAb and spike protein RBDs from non‐Omicron and Omicron variants, molecular dynamics simulations of D614G and Omicron BA.1 systems were carried out.

First, D614G and Omicron BA.1 systems were prepared, and missing atoms of residues were fixed based on the initial structure of D614G was retrieved from the RCSB Protein Data Bank (PDB code: 6XDG) by using PDBFixer and the tLEaP module in AmberTools 20.^[^ ^73^ ^]^

Second, each system was solvated by a cubic water box using the OPC water model with a margin of 10 Å. Periodic boundary condition (PBC) was used and the net charge was neutralized by Na+ ions. Nonbonded van der Waals interactions were calculated using the Lennard‐Jones 12–6 potentials with a 10 Å cutoff, while long‐range electrostatics were treated using the Particle Mesh Ewald (PME) algorithm.^[^ ^74^ ^]^ The SHAKE algorithm^[^ ^75^ ^]^ was applied to constrain bonds involving hydrogen atoms. To remove improper atom contacts, the structure was first minimized by^[^ ^1^ ^]^ 10000 steps of steepest descent and 10000 steps of conjugate gradient, under a harmonic constraint of 10.0 kcal/(mol·Å2) on heavy atoms;^[^ ^2^ ^]^ relaxing the entire system by 10000 steps of steepest descent and 10000 steps of conjugate gradient. And then the system was gradually heated up to 300 K by a 20 ps NVT simulation. Subsequently, two steps of equilibration phases were carried out:^[^ ^1^ ^]^ a 200 ps NPT simulation with constraints on heavy atoms followed by^[^ ^2^ ^]^ a 1 ns NVT simulation without restraint. The temperature was maintained at 300K using the Berendsen thermostat with 1 ps coupling constant and the pressure at 1 atm using Monte Carlo barostat with 1 ps relaxation time. Then, the system was subjected to a 100 ns NVT simulation with a time step of 2 fs. The binding free energies were calculated using the Molecular Mechanics Generalized Born (MM/PBSA)^[^ ^76^ ^]^ method for the 100 ns MD trajectory. The root‐mean‐square deviation (RMSD) and hydrogen bonds were analyzed using CPPTRAJ^[^ ^77^, ^78^ ^]^ module. And clustering was performed with the DBSCAN algorithm.^[^ ^79^ ^]^

## Conflict of Interest

The authors declare no conflict of interest.

## Author Contributions

X.Z., M.L., N.Z., Y.L., F.T., and Y.L. contributed equally to this work. X.Z., X.Z., X.X., and H.L. executed the experiments; Y.L., F.T., and Y.L. provided the clinical samples; M.L., X.Z., Y.Z.,Y.J., and Y.W. performed the data analysis; N.Z. and C.Q. provided mouse and human experimental samples and experimental data for pseudovirus and live virus‐based neutralization assays; X.Y., Y.W., S.G., and B.W. conceived the idea, designed experiments, and wrote the manuscript; X.Y., X.Z., and M.L. revised the manuscript.

## Supporting information

Supporting Information

## Data Availability

The data that support the findings of this study are openly available in iProX at https://www.iprox.cn, 9271000.
