# Supplementary material for: SARS‐CoV‐2 Evolution: Immune Dynamics, Omicron Specificity, and Predictive Modeling in Vaccinated Populations
Source: Adv Sci (Weinh). 2024 Aug 29;11(40):2402639. doi: 10.1002/advs.202402639 (PMC11516136; doi:10.1002/advs.202402639)
Supplement: Supplementary file 1 — Supporting Information [file ADVS-11-2402639-s001.docx]

- Supplementary Information -

**SARS-CoV-2 Evolution: Immune Dynamics, Omicron Specificity, and Predictive Modeling in Vaccinated Populations**

Xiaohan Zhang^1,2*^, Mansheng Li^1*^, Nana Zhang^4*^, Yunhui Li^3*^, Fei Teng^5*^， Yongzhe Li^6*^, Xiaomei Zhang^1^, Xingming Xu^1^, Haolong Li^6^, Yunping Zhu^1^, Yumin Wang^7^, Yan Jia^8^, Chengfeng Qin^4^, Bingwei Wang^2#^ , Shubin Guo^5#^, Yajie Wang^3#^ and Xiaobo Yu^1,7#^

**Affiliations**

^1^ State Key Laboratory of Medical Proteomics, Beijing Proteome Research Center, National Center for Protein Sciences-Beijing (PHOENIX Center), Beijing Institute of Lifeomics, Beijing, 102206, China..

^2^ School of Medicine, Nanjing University of Chinese Medicine, Nanjing 210023, China.

^3^ Department of Clinical Laboratory, Beijing Ditan Hospital, Capital Medical University, Beijing 100015, China.

^4^ Department of Virology, State Key Laboratory of Pathogen and Biosecurity, Beijing Institute of Microbiology and Epidemiology, Academy of Military Medical Sciences, Beijing, 100071, China.

^5^ Emergency Medicine Clinical Research Center, Beijing Chao-Yang Hospital, Capital Medical University, & Beijing Key Laboratory of Cardiopulmonary Cerebral Resuscitation, Beijing, 100020, China.

^6^ Department of Clinical Laboratory, Peking Union Medical College Hospital, Chinese Academy of Medical Science & Peking Union Medical College, Beijing 100730, China.

^7^ The First Affiliated Hospital of Wenzhou Medical University, Wenzhou, 325000, China.

^8^ ProteomicsEra Medical Co., Ltd., Beijing, 102206, China

^*^ These authors contributed equally to this work.

^#^ Correspondence to yuxiaobo@ncpsb.org.cn (X.Y.), wangyajie@ccmu.edu.cn (Y. W.), guoshubin@bjcyh.com (S. G.), bingweiwang@njucm.edu.cn (B. W.)

The supplementary contains nine figures and six tables.

**Supplementary Figures**


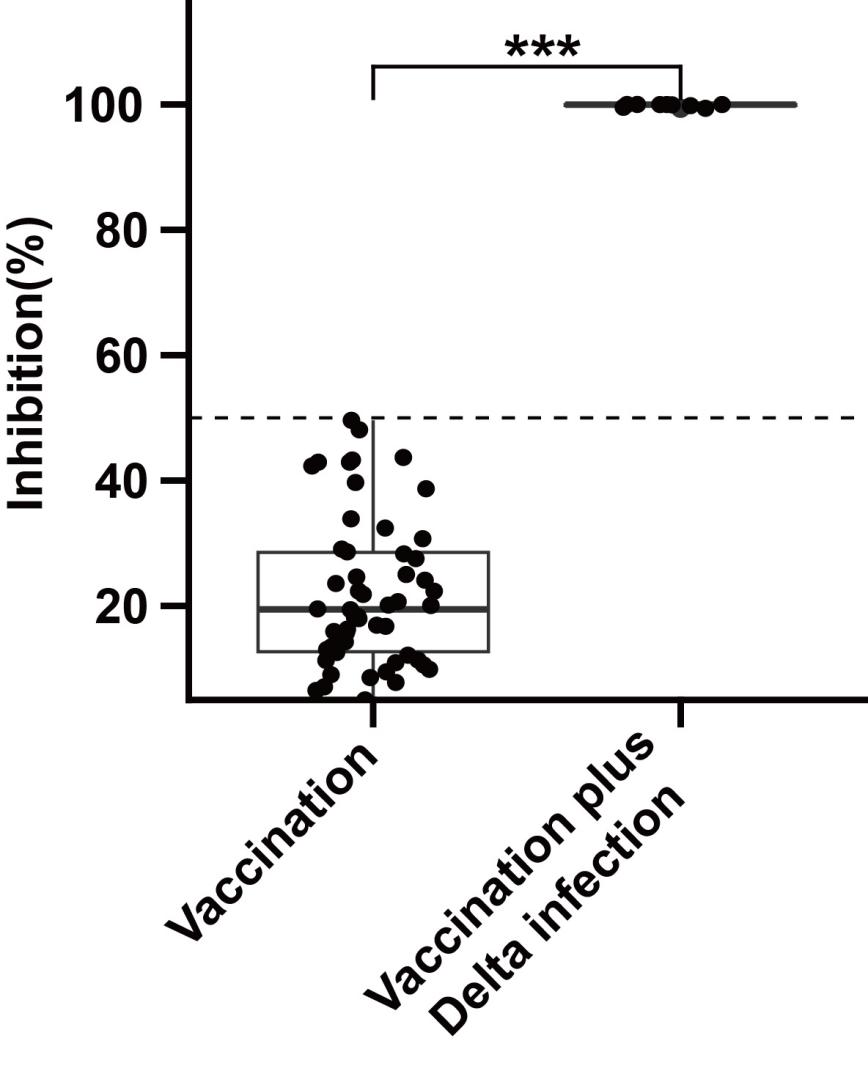


**Figure S1. Comparison of serum NAbs between** **COVID-19 vaccinated individuals and COVID-19 infected individuals.** Comparison of the differences in serum NAbs between COVID-19 vaccinated individuals (n = 54) and individuals with Delta variant breakthrough infection (n = 9), at 90 days after receiving the third dose of COVID-19 vaccine. The statistical analysis was performed using Wilcoxon rank-sum.test. *** represent p < 0.001.

**
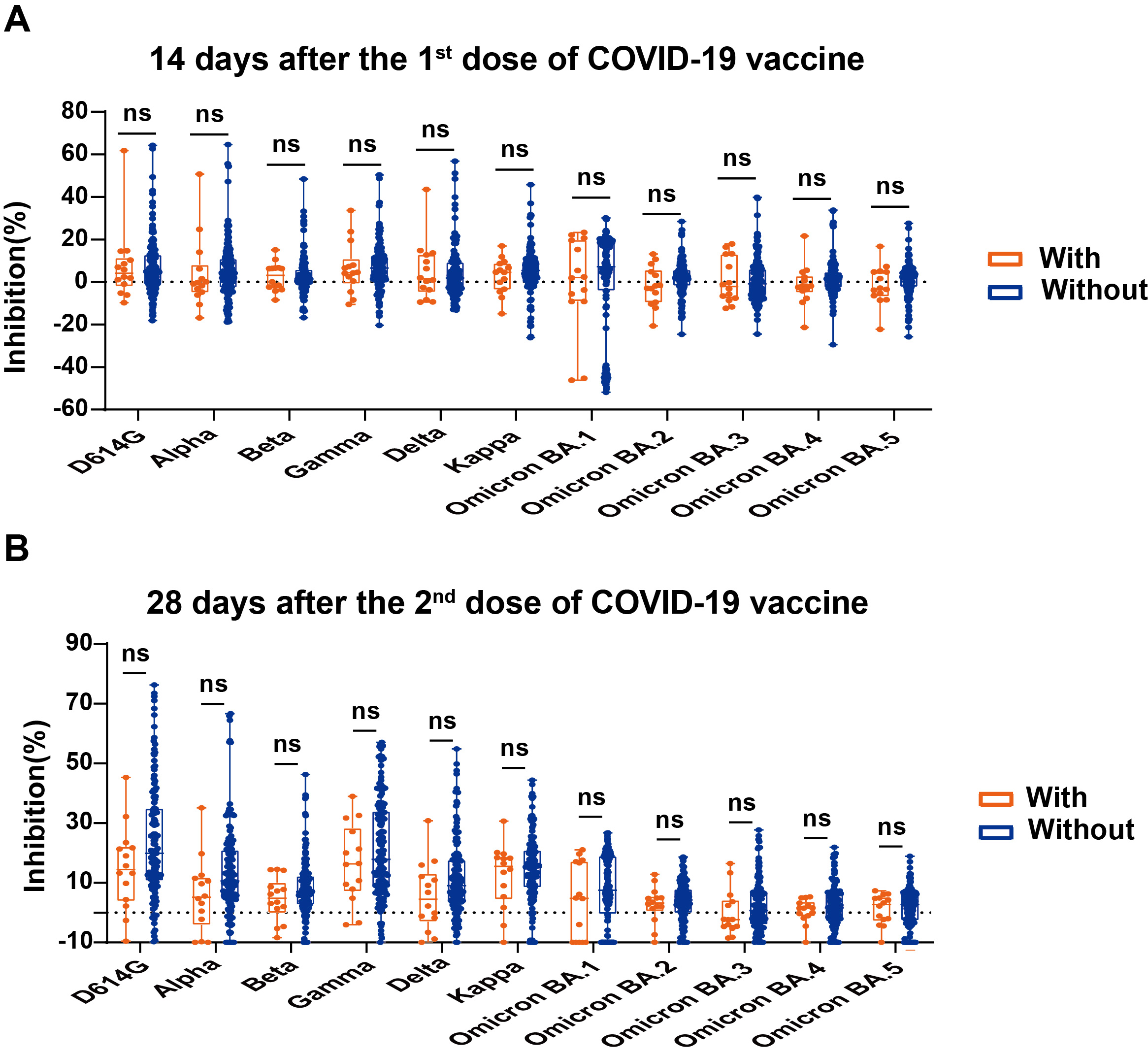
**

**Figure S2. Comparison of differences between groups with and without adverse effect of injection site pain, redness and swelling.** (A) The serum samples taken 14 days after the first dose of the COVID-19 vaccine were compared between the group that experienced the adverse effects (injection site pain, redness and swelling) and the group that did not. The results showed no significant difference between the two groups. (B) The serum samples taken 28 days after the second dose of the COVID-19 vaccine were compared between the group that experienced adverse effects (injection pain, redness and swelling) and the group that did not. The results showed no significant difference between the two groups. The comparison of differences between the two groups with and with no adverse effects were conducted using Multiple t-tests, ns represents p > 0.05.


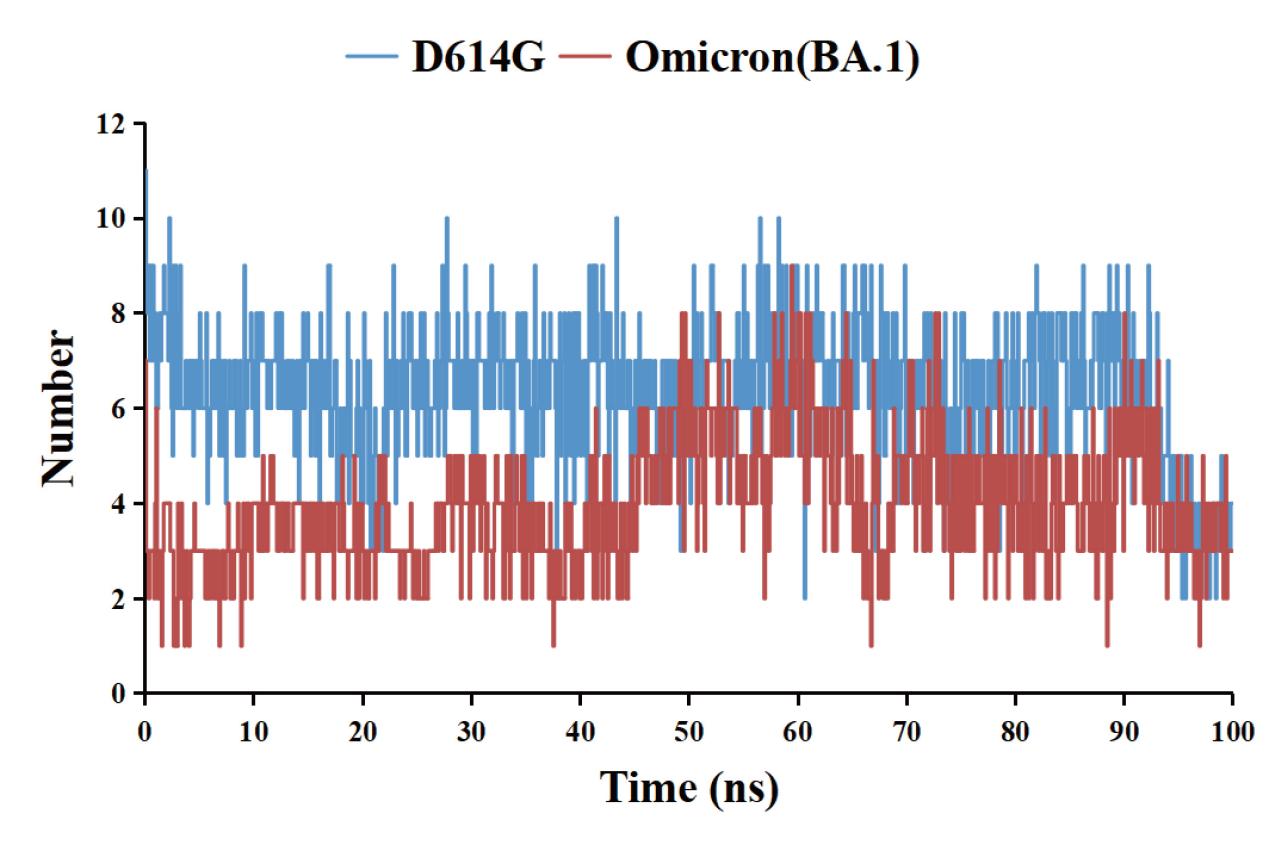


**Figure S3. Comparison of the structural stability of the D614G and Omicron BA.1 systems.** Plot of the number of hydrogen bonds for the D614G and Omicron BA.1 systems. Blue represents the D614G system, and red represents the Omicron BA.1 system.


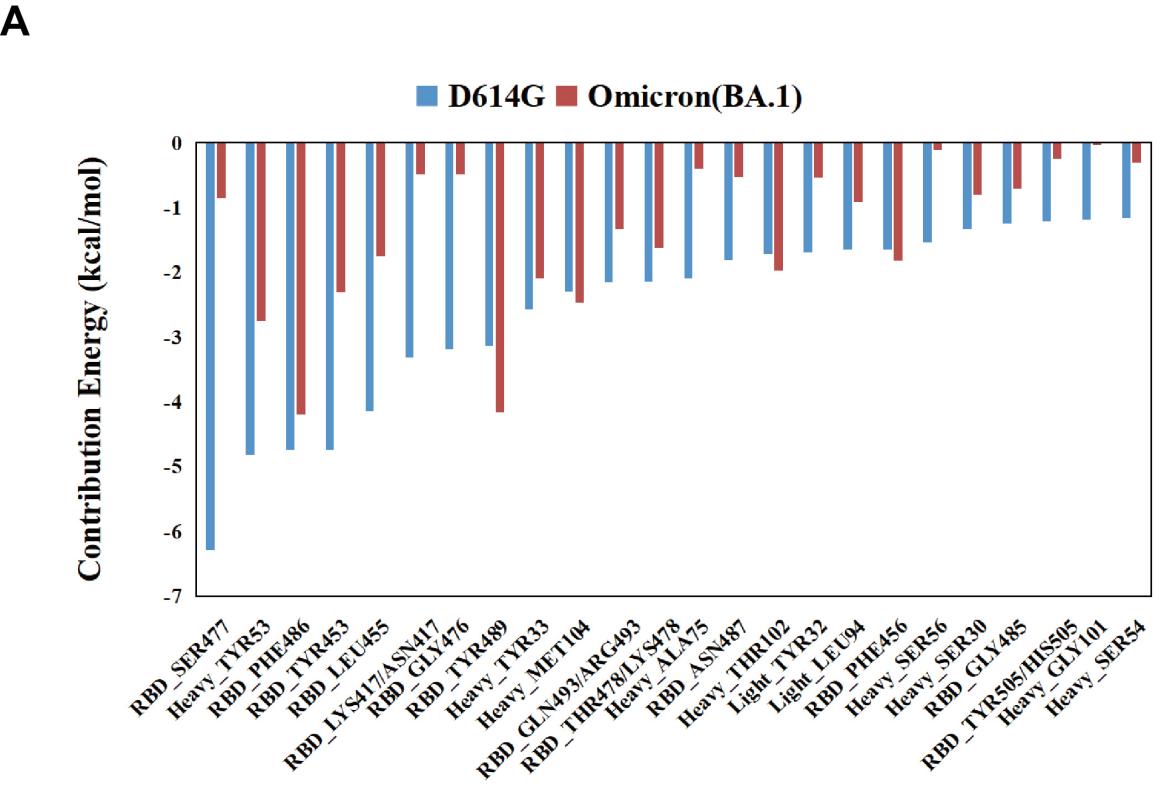


**Figure S4. Comparison of binding free energies between D614G and Omicron BA.1 systems.** Histogram of energy contribution decomposition of key residues of D614G and Omicron BA.1 systems. Blue represents the D614G system, and red represents the Omicron BA.1 system.

**
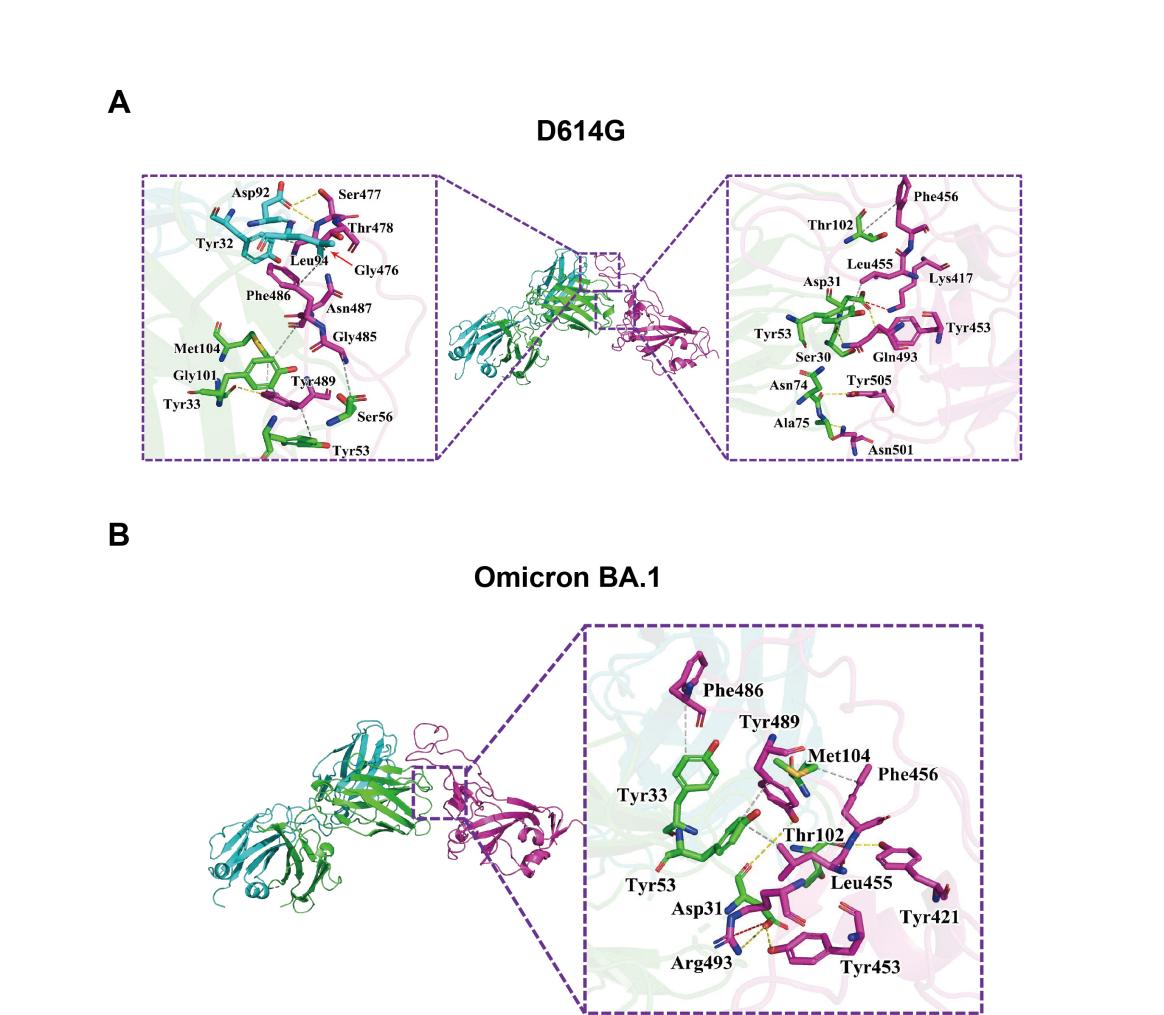
**

**Figure S5. Molecular dynamics cluster analysis of the D614G and Omicron BA.1 systems.** (A) Molecular dynamics cluster analysis of the D614G system: muants are in magenta cartoon, antibody heavy chain in green cartoon, antibody light chain in cyan cartoon; key residues are in magenta, green and blue-green stick; purple dash indicates the salt bridge action, yellow dash indicates the hydrogen bond action; gray dotted line indicates the hydrophobic action. (B) Molecular dynamics cluster analysis of the Omicron BA.1 system: muants are in magenta cartoon, antibody heavy chain in green cartoon, antibody light chain in cyan cartoon; key residues are in magenta, green and blue-green stick; purple dash indicates the salt bridge action, yellow dash indicates the hydrogen bond action; gray dotted line indicates the hydrophobic action.

**
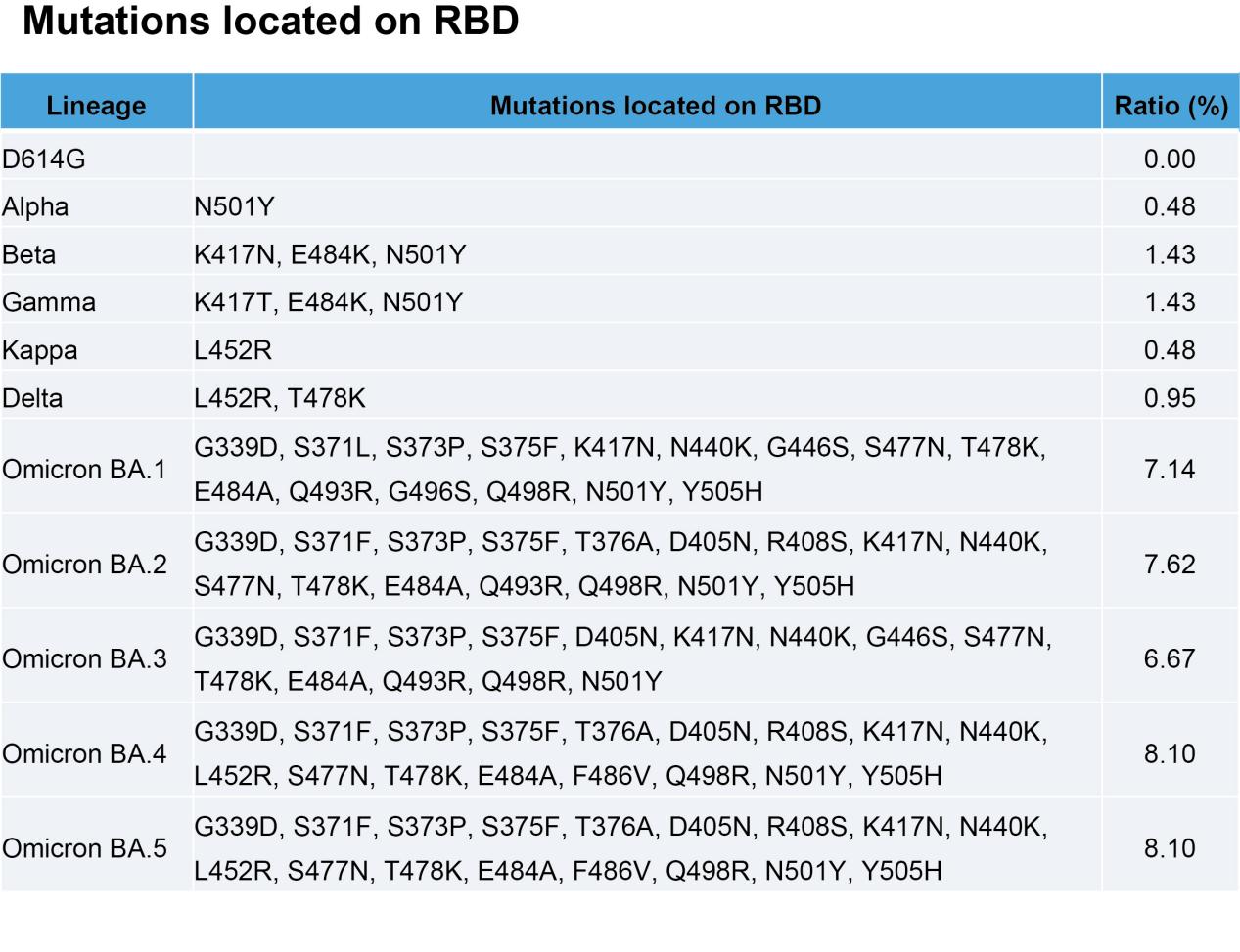
Figure S6. Amino Acid Mutations in the Receptor Binding Domain of SARS-CoV-2 Variants.** The percentage of mutated amino acids relative to the D614G variant.

**
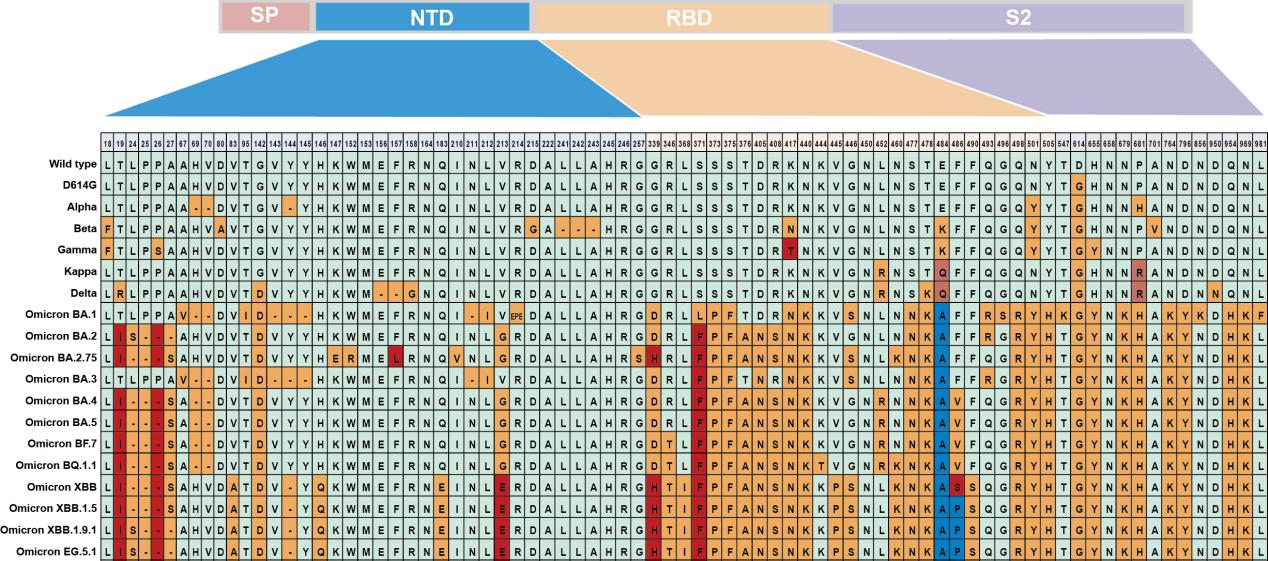
Figure S7. Visualization of Amino Acid Mutations in the Receptor Binding Domain (RBD) of the Spike Protein Across Different SARS-CoV-2 Variants from D614G to EG.5.1.**


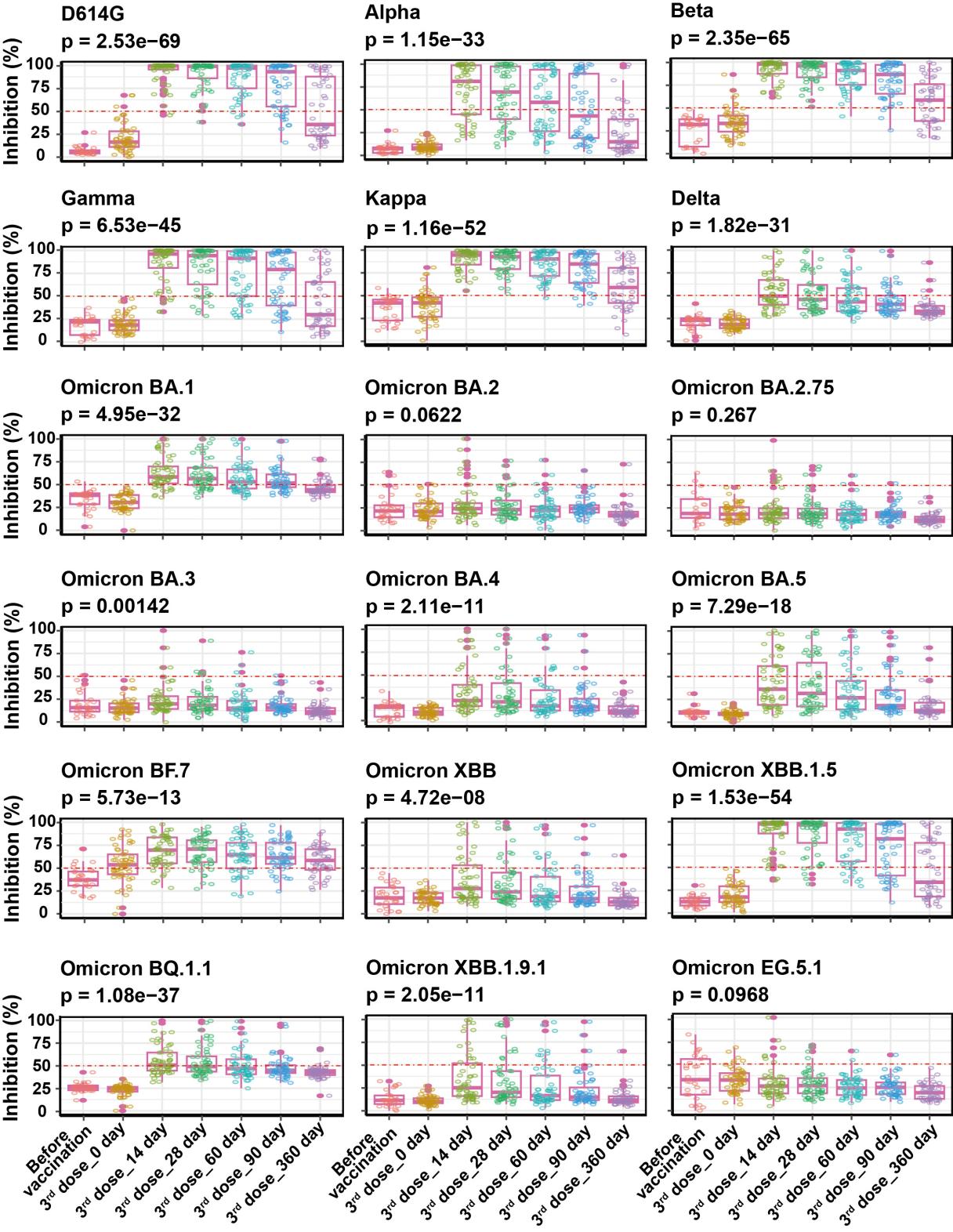


**Figure S8. Longitudinal Changes in the Inhibition Rate (%) of NAbs Against Different SARS-CoV-2 Variants in a Cohort Receiving a Third-Dose** **Inactivated or Recombinant Vaccine Administration.** The x-axis displays days before and after vaccination, and the y-axis depicts the inhibition rate (%) of serum antibodies after subtracting background levels obtained from pre-pandemic serum. The comparison of the mean of the inhibition rate (%) of NAbs against different SARS-CoV-2 variants were conducted using ANOVA test.

**
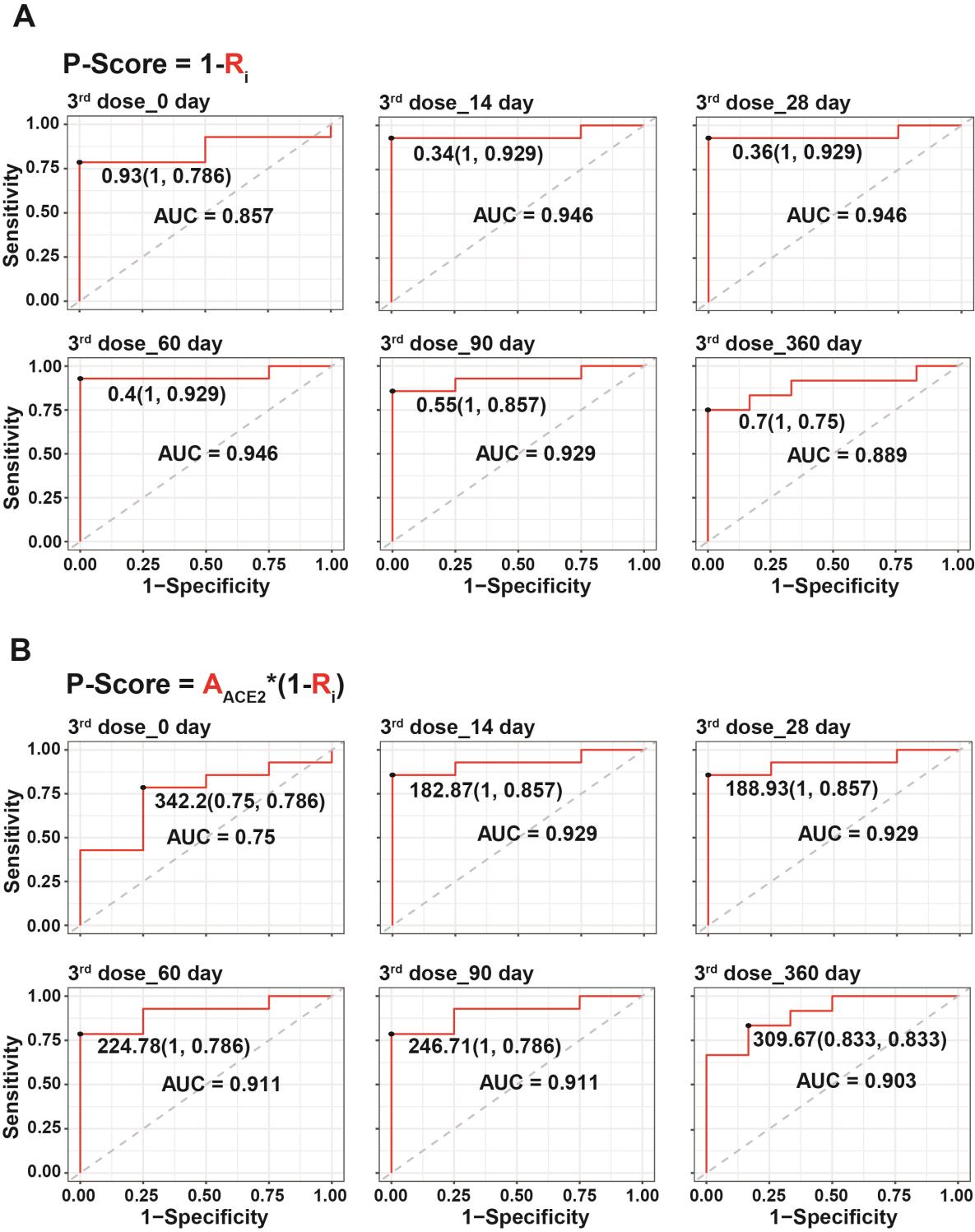
**

**Figure S9. Predictive Accuracy for Identifying SARS-CoV-2 Omicron Variants in the Inactivated or Recombinant Vaccine Administration Population.** (A) and (B) depict predictions of SARS-CoV-2 Omicron variants using serum NAbs without and with ACE2 binding, respectively. The AUCs were calculated using the pROC package (v 1.18.2), and optimal cutoff points were determined using the Youden index, implemented via the coords function in the pROC package.

**Supplementary Tables**

**Table S1. Demographics of COVID-19 vaccination cohort.**

|  | Chaoyang Cohort (n=441) | | | | Ditan Cohort (n=327) | | | | | | |
| --- | --- | --- | --- | --- | --- | --- | --- | --- | --- | --- | --- |
|  | Unvaccinated group | Vaccinated groups | |  | | Vaccinated groups | | | | | |
|  | (n=147) | 14 days after the 1^st^ dose  （n=147） | 28 days after the 2^nd^ dose  （n=147） |  | | 0 day after the 3^rd^ dose  (n=56) | 14 days after the 3^rd^ dose  (n=56) | 28 days after the 3^rd^ dose  (n=56) | 60 days after the 3^rd^ dose  (n=56) | 90 days after the 3^rd^ dose  (n=56) | 360 days after the 3^rd^ dose  (n=47) |
| Gender  Male  Female | 34(23.2%)  113(76.8%) | 34(23.2%)  113(76.8%) | 34(23.2%)  113(76.8%) |  | | 16(28.5%)  40(71.4%) | 16(28.5%)  40(71.4%) | 16(28.5%)  40(71.4%) | 16(28.5%)  40(71.4%) | 16(28.5%)  40(71.4%) | 14(29.7%)  33(70.2%) |
| Age(years)  Range  <50  ≥50 | 20-57  137(93.2%)  10(6.8%) | 20-57  137(93.2%)  10(6.8%) | 20-57  137(93.2%)  10(6.8%) |  | | 21-70  47(83.9%)  9(16.1%) | 21-70  47(83.9%)  9(16.1%) | 21-70  47(83.9%)  9(16.1%) | 21-70  47(83.9%)  9(16.1%) | 21-70  47(83.9%)  9(16.1%) | 22-70  39(83.0%)  8(17.0%) |
| **Vaccination type**  Recombinant  RBD-subunit vaccine (ZF2001)  Inactivated vaccine (Sinovac-CoronaVac) | /  / | 0  147(100%) | 0  147(100%) |  | | 38(83.9%)  18(16.1%) | 38(83.9%)  18(16.1%) | 38(83.9%)  18(16.1%) | 38(83.9%)  18(16.1%) | 38(83.9%)  18(16.1%) | 31(66.0%)  16(34.0%) |

**Table S2. List of adverse effects after COVID-19 first and second dose vaccination.**

|  | 14 days after the 1^st^ dose  （n = 147） | 28 days after the 2^nd^ dose  （n = 147） |
| --- | --- | --- |
| **Adverse effects**  Yes  No | 30 (20.4%)  117 (79.6%) | 23 (15.6%)  124 (84.4%) |
| **Number of adverse events per person**  0  1  2  3 | 117 (79.6%)  23 (15.0%)  5 (4.1%)  2 (1.4%) | 124 (84.4%)  19 (13.6%)  3 (1.4%)  1 (0.7%) |
| **Symptom**  Injection site pain, redness and swelling  Fatigue  Headache  Muscle pain  Induration  Pruritus  Cough  Dizziness  Joint pain  Nausea and vomiting  Throat pain  Array palpitations  Diarrhoea  Fever | 14 (37.8%)  3 (8.1%)  5 (13.5%)  5 (13.5%)  2 (5.4%)  2 (5.4%)  1 (2.7%)  2 (5.4%)  1 (2.7%)  1 (2.7%)  1 (2.7%)  0  0  0 | 14 (53.8%)  0  0  1 (3.8%)  3 (11.5%)  0  0  3 (11.5%)  0  1 (3.8%)  1 (3.8%)  1 (3.8%)  1 (3.8%)  1 (3.8%) |

**Table S3. Comparison of different high-throughput SARS-CoV-2 NAb detection methods****.**

| Technology | SARS-CoV-2 bNAb assay | Bead-based multiplex ACE2-RBD inhibition  assay (RBDCoV-ACE2) | Bio-Plex Pro Human SARS-CoV-2 Neutralization Antibody assay | FDA-approved cPass sVNT assay |
| --- | --- | --- | --- | --- |
| Platform | Wellgrow | Luminex | Bio-rad | ELISA |
| Number of targets per assay | Up to 100 | Up to 100 | Up to 100 | 1 |
| Spike protein type | Trimer | RBD | S1 and RBD | RBD |
| SARS-CoV-2 variants developed | Eighteen SARS-CoV-2 variants (D614G, Alpha, Beta, Gamma, Delta, Kappa, BA.1, BA.2, BA.3, BA.4, BA.5, BF.7, BA.2.75, BQ.1.1, XBB, XBB.1.5, XBB.1.9.1, and EG.5.1) | Twelve SARS-CoV-2 variants (Wild-type, Alpha, Beta, Gamma, Epsilon, Eta, Theta, Kappa, Delta, Lambda, Cluster 5 and A.23.1) | Eleven mutated S1 and RBD proteins, including Wild-type S1, Wild-type RBD, Alpha Spike 1, Beta Spike 1, Gamma RBD, Epsilon RBD, Kappa RBD, D614G Spike 1, E484K RBD, K417N RBD, and N501 RBD. | Wild-type |
| Reference | (*1*) | (*2*) | (*3*) | (*4*) |

**Table S4. Binding free energy of D614G and Omicron BA.1 system (unit: kcal/mol).**

| **Energy component** | **D614G** | **Omicron BA.1** |
| --- | --- | --- |
| ΔG_vdw_ | -104.9518±12.1806 | -76.4322±10.7946 |
| ΔG_ele_ | -216.4496±28.2791 | -137.4951±53.5378 |
| ΔG_polar_ | 257.8476±25.8651 | 174.7860±52.1602 |
| ΔG_nonpolar_ | -15.3114±1.7179 | -8.4851±0.8626 |
| ΔG_gas_ | -321.4013±33.1606 | -213.9273±52.7223 |
| ΔG_solv_ | 242.5362±24.9322 | 166.3009±51.8948 |
| ΔG_total_ | -78.8651±12.3103 | -47.6264±8.7390 |

ΔG_vdw_ is the van der Waals forces; ΔG_ele_ is the electrostatic forces; ΔG_polar_ is the polar solvation energy; ΔG_nonpolar_ is the nonpolar solvation energy; ΔG_gas_ is the molecular mechanics term (energy in the gas phase), ΔG_gas_ = ΔG_vdw_ + ΔG_ele_; ΔG_solv_ is the solvation energy, ΔG_solv_ = ΔG_polar_ + ΔG_nonpolar_; ΔG_total_ is the total binding free energy, ΔG_total_ = ΔG_gas_ + ΔG_solv_.

**Table S5. The time information of VOC/VOI obtained from the Nextrain (https://nextstrain.org/) and National Genomics Data Center (https://ngdc.cncb.ac.cn/ncov/monitoring/country/China).**

| Variant | 3^rd^ dose 0 day  Aug 2021 | 3^rd^ dose _14 days  Aug 2021 | 3^rd^ dose _28 days  Sep 2021 | 3^rd^ dose _60 days  Sep 2021 | 3^rd^ dose _90days  Nov 2021 | 3^rd^ dose _360 days  Aug 2022 |
| --- | --- | --- | --- | --- | --- | --- |
| D614G | 0 | 0 | 0 | 0 | 0 | 0 |
| Alpha | 0 | 0 | 0 | 0 | 0 | 0 |
| Beta | 0 | 0 | 0 | 0 | 0 | 0 |
| Gamma | 0 | 0 | 0 | 0 | 0 | 0 |
| Kappa | 0 | 0 | 0 | 0 | 0 | 0 |
| Delta | 1 | 1 | 1 | 1 | 1 | 0 |
| BA.1 | 1 | 1 | 1 | 1 | 1 | 1 |
| BA.2 | 1 | 1 | 1 | 1 | 1 | 1 |
| BA.2.75 | 1 | 1 | 1 | 1 | 1 | 1 |
| BA.3 | 1 | 1 | 1 | 1 | 1 | 1 |
| BA.4 | 1 | 1 | 1 | 1 | 1 | 1 |
| BA.5 | 1 | 1 | 1 | 1 | 1 | 1 |
| BF.7 | 1 | 1 | 1 | 1 | 1 | 1 |
| BQ.1.1 | 1 | 1 | 1 | 1 | 1 | 1 |
| XBB | 1 | 1 | 1 | 1 | 1 | 1 |
| XBB.1.5 | 1 | 1 | 1 | 1 | 1 | 1 |
| XBB.1.9.1 | 1 | 1 | 1 | 1 | 1 | 1 |
| EG.5.1 | 1 | 1 | 1 | 1 | 1 | 1 |

**Table S6. Summary of COVID-19 vaccines employed in this work.**

| **Type of vaccine** | WT inactivated vaccine | WT recombinant  RBD vaccine | WT mRNA vaccine | Delta mRNA  vaccine | Omicron BA.4/5 mRNA vaccine |
| --- | --- | --- | --- | --- | --- |
| **SARS-CoV-2 variant** | WT | WT | WT | Delta | Omicron BA.4/5 |
| **Antigen type** | Whole virus | S-RBD protein | S-RBD protein | S-RBD protein | S-RBD protein |
| **Vaccine manufacturer** | Sinovac Biotech | Anhui Zhifei Longcom | Laboratory-made | Laboratory-made | Laboratory-made |
| **Reference** | (*5*) | (*6*) | (*7*) | (*7*) | (*8*) |

**Reference**

1. X. Zhang *et al.*, High-throughput detection of neutralizing antibodies to SARS-CoV-2 variants using flow cytometry. 2023.2008.2007.23293304 (2023).

2. D. Junker *et al.*, COVID-19 patient serum less potently inhibits ACE2-RBD binding for various SARS-CoV-2 RBD mutants. *Sci Rep* **12**, 7168 (2022).

3. A. L. Heaps *et al.*, Rapid determination of SARS-CoV-2 antibody neutralization titer using Bio-Rad Bio-Plex correlates strongly with pseudovirus-determined neutralization titer. *J Virol Methods* **316**, 114726 (2023).

4. C. W. Tan *et al.*, A SARS-CoV-2 surrogate virus neutralization test based on antibody-mediated blockage of ACE2-spike protein-protein interaction. *Nat Biotechnol* **38**, 1073-1078 (2020).

5. Z. Wu *et al.*, Safety, tolerability, and immunogenicity of an inactivated SARS-CoV-2 vaccine (CoronaVac) in healthy adults aged 60 years and older: a randomised, double-blind, placebo-controlled, phase 1/2 clinical trial. *Lancet Infect Dis* **21**, 803-812 (2021).

6. S. Yang *et al.*, Safety and immunogenicity of a recombinant tandem-repeat dimeric RBD-based protein subunit vaccine (ZF2001) against COVID-19 in adults: two randomised, double-blind, placebo-controlled, phase 1 and 2 trials. *Lancet Infect Dis* **21**, 1107-1119 (2021).

7. N. N. Zhang *et al.*, A Thermostable mRNA Vaccine against COVID-19. *Cell* **182**, 1271-1283 e1216 (2020).

8. H. Zhao *et al.*, Neutralization of Omicron XBB.1 by booster vaccination with BA.4/5 monovalent mRNA vaccine. *Cell Discov* **10**, 7 (2024).
